# Supplementary material for: Molecular basis of Fab-dependent IgA antibody recognition by gut-bacterial metallopeptidases
Source: EMBO J. 2025 Jul 31;44(17):4867–98. doi: 10.1038/s44318-025-00518-w (PMC12402451; doi:10.1038/s44318-025-00518-w)
Supplement: Supplementary file 1 — Appendix [file 44318_2025_518_MOESM1_ESM.pdf]

Appendix for:

Molecular basis of Fab-dependent IgA antibody recognition by gut-bacterial metallopeptidases

María Ángeles Márquez-Moñino[1#], Ana Martínez Gascueña[1#], Tala Azzam[2], Andrea Persson[3], Aitor Manzanares-Gomez[1], Marina Aguillo-Urarte[1], Trenton T. Brown[2], Ainhoa Montero-Sagarminaga[1], Rolf Lood[3], Andreas Naegeli[3], Sean R. Connell[4,5], Diego E. Sastre[2], Eric J. Sundberg[2] and Beatriz Trastoy[1,5]*

[#]These authors contributed equally

[1]Structural Glycoimmunology Laboratory, Biobizkaia Health Research Institute, Barakaldo, Spain.

[2]Department of Biochemistry, Emory University School of Medicine, Atlanta, GA, USA. [3] Genovis AB, Box 790, 22007, Lund, Sweden.

[4]Structural Biology of Cellular Machines Laboratory, Biobizkaia Health Research Institute, Barakaldo, Spain.

[5]Ikerbasque, Basque Foundation for Science, 48009 Bilbao, Spain.

Table of Contents

1. *Appendix Tables* 3

Appendix Table S1. Sequence of primers used to generate ThomasA, BF3526 and IgA1 constructs and mutants. 3

Appendix Table S2. SAXS data and refinement statistics. 5

Appendix Table S3. Data collection and refinement statistics of ThomasA$^{323-878}$ and BF3526 X-ray structures. 6

Appendix Table S4. Root mean squared deviation (r.m.s.d.) of protein molecules in the crystal structure of BF3526. 8

2. *Appendix Figures* 9

Appendix Figure S1. Sequence conservation of the catalytic motif in the M64 peptidase family. 9

Appendix Figure S2. Structural homologues of ThomasA domains. 10

Appendix Figure S3. SAXS analysis of ThomasA constructs 11

Appendix Figure S4. ThomasA$^{31-878}$ negative stain (NS) reconstruction 12

Appendix Figure S5. Intact LC-MS hydrolytic activity assays of ThomasA and IgA1 constructs. 13

Appendix Figure S6. Hydrolytic activity of ThomasA constructs against $IgA1_{fl}$, MBP-TEV-$IgA1_{HR}$-EGFP, and $IgG1_{Fab}$-$IgA1_{HR-Fc}$. 14

Appendix Figure S7. Recombinant production of ThomasA$^{323-878}$ and BF3526. 15

Appendix Figure S8. Electron density maps of the refined ThomasA$^{323-878}$ X-ray crystal structure. 16

Appendix Figure S9. SAXS analysis of ThomasA$^{323-878}$. 17

Appendix Figure S10. Hydrolytic activity of BF3526 towards predigested hIgA2 by IgASAP Sub1+2. 18

Appendix Figure S11. Hydrolytic activity of BF3526 towards hIgA2 and IgG1 peptides analyzed by LC-MS/MS. 19

Appendix Figure S12. Electron density maps of the refined BF3526 X-ray crystal structure. 20

Appendix Figure S13. Electron density maps of the active site of BF3526-unliganded, BF3526-STPP and BF3526-PP molecules. 21

Appendix Figure S14. Structural homologues of BF3526 domains. 22

Appendix Figure S15. Catalytic mechanism of BF3526. 23

Appendix Figure S16. Hydrolytic activity of $BF3526_{NTD}$, $BF3526_{CT1}$ and $BF3526_{CT2}$ against IgA1, IgA2 and IgG1 24

Appendix Figure S17 | Analysis by size exclusion and SDS-PAGE of ThomasA$^{323-878}$ and $IgA1_{Fab-HR}$ complex formation. 25

3. *Appendix text* *26*

Structural homologues of ThomasA individual domains 26

Structural homologues of BF3526 26

4. *References* *27*

1. Appendix Tables

Appendix Table S1. Sequence of primers used to generate ThomasA, BF3526 and IgA1 constructs and mutants.

Protein	Primer sense	Primer sequence 5'-3'	PCR template vector
**ThomasA$^{31-1167}$**	***pGEX-4T-1 (ATG:biosynthetics GmbH)***		
ThomasA$^{31-878}$	Forward	cagatggcaccgtggttacttactactacaagaacaaaaattgactcga	ThomasA$^{31-1167}$
	Reverse	gtcacgatgcggccgctcgagtcaatttttgt	
ThomasA$^{31-795}$	Forward	gacacggtagcgttccaagtgctggatgaaaactgactcga	ThomasA$^{31-1167}$
	Reverse	gtcacgatgcggccgctcgagtcagttttcat	
ThomasA$^{31-632}$	Forward	agggctttaaacgcatgtcccagctggtgaaatgactcga	ThomasA$^{31-1167}$
	Reverse	gtcacgatgcggccgctcgagtcatttcacca	
ThomasA$^{323-632}$	Forward	cctttacttccagggcggcagcggcccggactg	ThomasA$^{31-632}$
	Reverse	ggactgttcggtgctcacattccagtccgggccgctgc	
ThomasA$^{323-878}$	Forward	cctttacttccagggcggcagcggcccggactg	ThomasA$^{31-878}$
	Reverse	ggactgttcggtgctcacattccagtccgggccgctgc	
ThomasA$^{323-878}$-W340A	Forward	caccctgggcccggcgtacagcaatgatg	ThomasA$^{323-878}$
	Reverse	catcattgctgtacgccgggcccagggtg	
ThomasA$^{323-878}$-K435A	Forward	cttcgacgtaatcgtggatgcatataactctccggtgatcagc	ThomasA$^{323-878}$
	Reverse	gctgatcaccggagagttatatgcatccacgattacgtcgaag	
ThomasA$^{323-878}$-Y436A	Forward	gacgtaatcgtggataaggcaaactctccggtgatcagcaac	ThomasA$^{323-878}$
	Reverse	gttgctgatcaccggagagtttgccttatccacgattacgtc	
ThomasA$^{323-878}$-N437A	Forward	gtaatcgtggataagtatgcatctccggtgatcagcaacaacc	ThomasA$^{323-878}$
	Reverse	ggttgttgctgatcaccggagatgcatacttatccacgattac	
ThomasA$^{323-878}$-N444A	Forward	ctccggtgatcagcaacgcactccatggttctcagtg	ThomasA$^{323-878}$
	Reverse	cactgagaaccatggagtgcgttgctgatcaccggag	
ThomasA$^{323-878}$-W450A	Forward	caacaacctccatggttctcaggcaaaaaaccacattttcgagcg	ThomasA$^{323-878}$
	Reverse	cgctcgaaaatgtggttttttgcctgagaaccatggaggttgttg	
ThomasA$^{323-878}$-H453A	Forward	catggttctcagtggaaaaacgcaattttcgagcgttgcatcg	ThomasA$^{323-878}$
	Reverse	cgatgcaacgctcgaaaattgcgtttttccactgagaaccatg	
ThomasA$^{323-878}$-E486A	Forward	ccttccggttctgcatacgagccgtactac	ThomasA$^{323-878}$
	Reverse	gtagtacggctcgtatgcagaaccggaagg	
ThomasA$^{323-878}$-Y487A	Forward	catcccttccggttctgaagcagagccgtactactatgtac	ThomasA$^{323-878}$
	Reverse	gtacatagtagtacggctctgcttcagaaccggaagggatg	
ThomasA$^{323-878}$-N516A	Forward	ctttggcggcgcatataacgcacgtgagtatggcttcc	ThomasA$^{323-878}$
	Reverse	ggaagccatactcacgtgcgttatatgcgccgccaaag	
ThomasA$^{323-878}$-Y519A	Forward	gcgcatataacaaccgtgaggcaggcttccactatttcatttc	ThomasA$^{323-878}$
	Reverse	gaaatgaaatagtggaagcctgcctcacggttgttatatgcgc	
ThomasA$^{323-878}$-H539A/E540A	Forward	ccgtgcttctaaaactttcgcggctgcattcggtcacggtctcc	ThomasA$^{323-878}$
	Reverse	ggagaccgtgaccgaatgcagccgcgaaagttttagaagcacgg	
ThomasA$^{323-878}$-K561A	Forward	gctacctgctggacgacgcagaattgaaaagcctg	ThomasA$^{323-878}$
	Reverse	caggcttttcaattctgcgtcgtccagcaggtagc	
**BF3526**	***pSpeedET (DNASU Plasmid Repository)***		
BF3526$^{161-426}$	Forward	tccagggcggcactccgcacaagtatctgctgaaaagtg	BF3526
	Reverse	ggagtgccgccctggaagtacaggttttcgtgatgatgatgatg	
**IgA1$_{fl}$**	***pcDNA3.1(+) (ATG:biosynthetics GmbH)***		
IgA1$_{fl}$-K126A	Forward	ctcccacttctcccgctgtgttccctctgagcc	IgA1$_{fl}$
	Reverse	ggctcagagggaacacagcgggagaagtgggag	
IgA1$_{fl}$-S131A	Forward	caaggtgttccctctggctctctgtagcacccagc	IgA1$_{fl}$
	Reverse	gctgggtgctacagagagccagagggaacaccttg	
IgA1$_{fl}$-L132A	Forward	gtgttccctctgagcgcttgtagcacccagcccg	IgA1$_{fl}$
	Reverse	cgggctgggtgctacaagcgctcagagggaacac	

Appendix Table S1 (continued)

Protein (heavy chain)		Primer sense	Primer sequence 5'-3'	PCR template vector
IgA1$_{fl}$-S161A		Forward	ctcagcgtcacatgggctgagagtggacagg	IgA1$_{fl}$
		Reverse	cctgtccactctcagcccatgtgacgctgag	
IgA1$_{fl}$-D181A		Forward	gacgcctctggggctctgtacactacc	IgA1$_{fl}$
		Reverse	ggtagtgtacagagccccagaggcgtc	
IgA1$_{fl}$-T194A		Forward	gaccctgcctgcagctcagtgtctcg	IgA1$_{fl}$
		Reverse	cgagacactgagctgcaggcagggtc	
IgA1$_{fl}$-Q195A		Forward	gaccctgcctgcaactgcttgtctcgccggaaaatc	IgA1$_{fl}$
		Reverse	gattttccggcgagacaagcagttgcaggcagggtc	
IgA1$_{fl}$-K200A		Forward	cagtgtctcgccggagcttccgtcacatgccac	IgA1$_{fl}$
		Reverse	gtggcatgtgacggaagctccggcgagacactg	
IgA1$_{fl}$-S201A		Forward	gtgtctcgccggaaaagctgtcacatgccacgtgaag	IgA1$_{fl}$
		Reverse	cttcacgtggcatgtgacagcttttccggcgagacac	
IgA1$_{fl}$-H205A		Forward	cggaaaatccgtcacatgcgctgtgaagcactacactaatcc	IgA1$_{fl}$
		Reverse	ggattagtgtagtgcttcacagcgcatgtgacggattttccg	
IgA1$_{fl}$-N211A		Forward	gtgaagcactacactgctccaagccaggacgtg	IgA1$_{fl}$
		Reverse	cacgtcctggcttggagcagtgtagtgcttcac	
IgA1$_{fl}$-P212S		Forward	gtgaagcactacactaattccagccaggacgtgactgtcc	IgA1$_{fl}$
		Reverse	ggacagtcacgtcctggctggaattagtgtagtgcttcac	
IgA1$_{fl}$-S213A		Forward	gaagcactacactaatccagctcaggacgtgactgtccc	IgA1$_{fl}$
		Reverse	gggacagtcacgtcctgagctggattagtgtagtgcttc	
IgA1$_{fl}$-D215A		Forward	ctaatccaagccaggctgtgactgtcccctgc	IgA1$_{fl}$
		Reverse	gcaggggacagtcacagcctggcttggattag	
IgA1$_{fl}$-C220S		Forward	gtgactgtcccctcacctgtcccaagcacac	IgA1$_{fl}$
		Reverse	gtgtgcttgggacaggtgaggggacagtcac	
IgA1$_{Fab\text{-}HR}$		Forward	accaccactaatctagagggcccgtttaaacccgctgatca	IgA1$_{fl}$
		Reverse	ctagattagtggtggtggtggtggtggcttcctgatgggctgggtgtgggtggggttgagg	
IgA1$_{Fab\text{-}PVPS}$		Forward	accaccactaatctagagggcccgtttaaacccgctgatca	IgA1$_{fl}$
		Reverse	ctagattagtggtggtggtggtggtggcttgggacagggcaggggacagtcacg	
IgA1$_{Fab\text{-}HR}$ - IgG1$_{Fc}$	Insert	Forward	tacattcccagctccagctccaggaaagcggccca	IgA1$_{fl}$
		Reverse	ggtccccctgatgggctgggtgtgggtggggttgagggag	
	Vector	Forward	gcccatcagggggaccgtcagtcttcctcttccccca	IgG1 *pN12-i3*
		Reverse	tggagctgggaatgtacaccggtggcagttgctactagaaaaag	
IgG1$_{Fab}$ - IgA1$_{HR\text{-}Fc}$	Insert	Forward	aatcttgtcctgtcccaagcacacccccaactccttc	IgG1
		Reverse	gccgctcacttcccggcgagcctgtcaatggtcttc	
	Vector	Forward	ccgggaagtgagcggccgcaagcttggcc	IgA1$_{fl}$
		Reverse	gggacaggacaagatttgggctcaactctcttgtccaccttgg	
**MBP-TEV-EGFP**			***pMBP-TEV-EGFP***	
MBP-TEV-IgA1$_{HR}$-EGFP		Forward	actccttctccctcaaccccaccgacacctagcccatcaggctccggtgtgagcaagggcga ggagctgttcacc	*MBP-TEV-GFP*
		Reverse	ttgagggagaaggagttggaggtgtgcttgggacagggcagggcgacccgccctggaagt acaggttttcgccgctg	

Appendix Table S2. SAXS data and refinement statistics.

	ThomasA$^{31-1167}$	ThomasA$^{31-878}$	ThomasA$^{323-878}$
**SASBDB accession code**	SASDX45	SASDX55	SASDX65
**Data collection**			
Beamline	B21 (DLS)	B21 (DLS)	B21 (DLS)
Collection date	2024-04-19	2024-04-19	2024-04-19
Detector	EigerX 4M	EigerX 4M	EigerX 4M
Temperature (K)	288	288	288
Wavelength (Å)	0.9464	0.9464	0.9464
Energy (keV)	13.1	13.1	13.1
S range ($Å^{-1}$)	0.002 - 0.420	0.002 - 0.420	0.012 - 0.044
Q range ($Å^{-1}$)(1)	0.0045 - 0.34	0.0045 - 0.34	0.0045 - 0.34
Exposure time (s per frame)	0.56	0.56	0.56
Concentration range (mM)	16	20	46
**Structure parameters**			
I(0) (a.u.)(2) (from *P(r)*)	0.048	0.043	0.031
R_g (Å) (from *P(r)*)	63.78	34.57	29.6
I(0) (a.u.)(2) (from Guinier)	0.048	0.043	0.031
R_g (Å) (from Guinier)	64.43	34.43	29.52
D_{max} (Å)	250	106	92
Porod volume estimate ($Å^3$)	207,699	100,959	72,087
Ab initio modeling (χ^2 value)	1.4	2.1	1.6
**Molecular mass determination**			
Predicted molecular weight (kDa)(3)	155.1	91.7	69.2
Calculated MW from sequence (kDa)(4)	127.1	96.3	63.7
Discrepancy (%)	22.0	4.8	8.6
Oligomeric state	monomer	monomer	monomer
**Software employed**			
Primary data reduction	*SCÅTTER*	*SCÅTTER*	*SCÅTTER*
Data processing	*SCÅTTER, PRIMUS*	*SCÅTTER, PRIMUS*	*SCÅTTER, PRIMUS*

(1) *Q definition is* $4\pi*\sin(\theta)/\lambda$
(2) *arbitrary unit*
(3) *Molecular weight determination by SAXSMoW by SAXS data*
(4) *Molecular weight determination by ProtParam (EXPASY) by protein sequence*

Appendix Table S3. Data collection and refinement statistics of ThomasA$^{323-878}$ and BF3526 X-ray structures.

	ThomasA$^{323-878}$	BF3526
PDB accession codes	9QDH	9QDI
Space group	$P2_1 2_1 2_1$	P1
**Unit cell parameters**		
a, b, c (Å)	70.74, 85.20, 90.67	99.29, 99.44, 103.39
α, β, γ, (°)	90, 90, 90	74.16, 88.13, 82.44
Molecules per ASU	1	8
Matthews Coefficient (% solvent)	2.14 (42.68)	2.64 (53.43)
**Data collection**		
Beamline	i24 (DLS)	i24 (DLS)
Collection date	2024-04-27	2024-02-01
Temperature (K)	100	100
Wavelength (Å)	0.6199	0.9786
Resolution (Å)	45.33 - 2.00 (2.07 - 2.00)	29.64 - 1.94 (1.96 - 1.94)
**Data processing**		
Total reflections	507,392 (46,752)	945,575 (29,219)
Unique reflections	37,731 (3,740)	271,117 (8,246)
Multiplicity	13.4 (12.5)	3.5 (3.5)
Completeness (%)	99.95 (100.00)	96.95 (88.79)
Mean I/σ (I)	5.91 (0.83)	8.67 (1.59)
Wilson B-factor (Å^2)	31.57	33.99
R_{merge}	0.346 (2.869)	0.078 (0.939)
R_{meas}	0.360 (2.993)	0.093 (1.109)
R_{pim}	0.098 (0.847)	0.050 (0.587)
$CC_{1/2}$	0.994 (0.534)	0.997 (0.425)
CC*	0.999 (0.835)	0.999 (0.772)
**Refinement**		
Reflections used in refinement	37,717 (3,740)	271,069 (8,267)
Reflections used for R_{free}	1,807 (176)	13,524 (415)
R_{work}	0.1941 (0.3350)	0.1594 (0.2719)
R_{free}	0.2448 (0.3746)	0.1976 (0.3215)
CC_{work}	0.966 (0.728)	0.965 (0.829)
CC_{free}	0.941 (0.713)	0.957 (0.733)
**Number atoms (Average B-factor, Å^2)**		
Non-hydrogen atoms	4,757 (35.84)	27,342 (42.65)
Macromolecules	Chain A 4,490 (35.74)	Chain A 3,213 (35.17)<br>Chain B 3,214 (37.79)<br>Chain C 3,245 (36.29)<br>Chain D 3,225 (40.39)<br>Chain E 3,221 (39.59)<br>Chain F 3,214 (39.55)<br>Chain G 3,205 (51.90)<br>Chain H 3,205 (59.63)
Substrates/Products	0 (0.00)	Chain I product 22 (45.05)<br>Chain J substrate 28 (61.18)<br>Chain K substrate 28 (50.70)<br>Chain L product 22 (54.14)
Zn^{2+} ions	Catalytic 0 (0.00)<br>Secondary 1 (32.87)	Catalytic unliganded 4 (44.57)<br>Catalytic with substrate 2 (62.83)<br>Catalytic with product 2 (58.01)<br>Secondary 8 (36.86)

Appendix Table S3 (continued)

	ThomasA$^{323-878}$	BF3526
Water molecules	259 (37.87)	1,375 (41.62)
Protein residues built	Chain A 323 - 876	Chain A 21-327, 332-426<br>Chain B 21-328, 333-426<br>Chain C 21-426<br>Chain D 21-330, 334-426<br>Chain E 21-327, 331-426<br>Chain F 21-328, 333-426<br>Chain G 21-327, 333-426<br>Chain H 21-327, 333-426
**Geometry**		
RMS-bonds (Å)	0.003	0.008
RMS-angles (º)	0.623	0.90
Ramachandran favoured (outliers) (%)	96.74 (0.00)	97.50 (0.00)
Rotamer outliers (%)	0.60	1.12
Clashscore	2.73	2.23

Statistics for the highest resolution shell are shown in parenthesis.

Appendix Table S4. Root mean squared deviation (r.m.s.d.) of protein molecules in the crystal structure of BF3526.

BF3526-unliganded (chains A, B, G and H), BF3526-PP (chains C and F) and BF3526-STPP (chains D and E) molecules.

Chain	A	B	C	D	E	F	G	H
A		0.164	0.576	0.618	0.561	0.602	0.307	0.317
B	0.164		0.547	0.582	0.545	0.566	0.293	0.286
C	0.576	0.547		0.241	0.138	0.232	0.472	0.495
D	0.618	0.582	0.241		0.246	0.110	0.514	0.518
E	0.561	0.545	0.138	0.246		0.236	0.452	0.479
F	0.602	0.566	0.232	0.110	0.236		0.502	0.517
G	0.307	0.293	0.472	0.514	0.452	0.502		0.218
H	0.317	0.286	0.495	0.518	0.479	0.517	0.218	

2. Appendix Figures

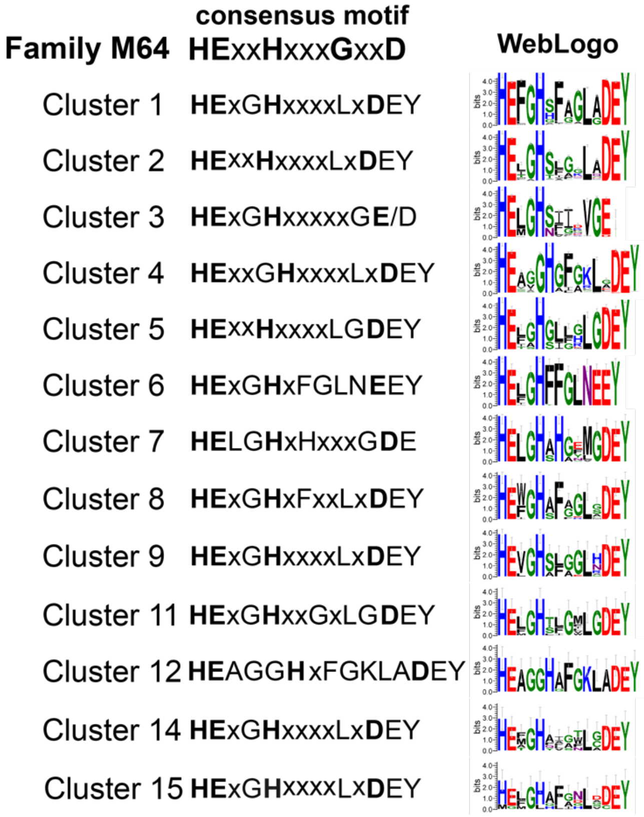

Appendix Figure S1. Sequence conservation of the catalytic motif in the M64 peptidase family. Sequence logo (obtained using WebLogo: https://weblogo.berkeley.edu/logo.cgi) representing the multiple sequence alignment of the consensus catalytic motif of M64 peptidase family corresponding to each cluster of our sequence similarity network analysis (SSN).

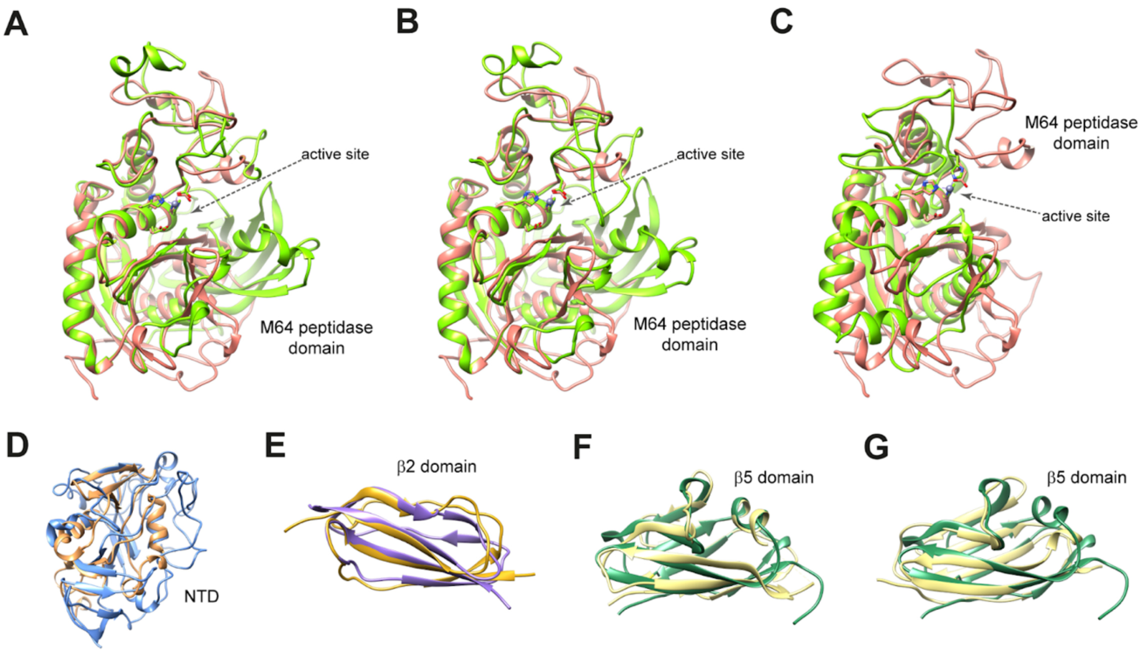

Appendix Figure S2. Structural homologues of ThomasA domains.

Structural superposition of X-ray structures of DALI homologues from PDB database with AF3 domains of ThomasA. **A-C** M64 peptidase domain of ThomasA (salmon) and BF3526 M64 peptidase from *Bacteroides fragilis* (PDB code 4DF9, chain F, green) (**A**), BACOVA_00663 M64 peptidase from *B. ovatus* (PDB code 3P1V, chain A, green) (**B**) and leucurolysin M12 peptidase from *Bothrops leucurus* venom (PDB code 4Q1L, chain A, green) (**C**). **D** ThomasA NTD (blue) and collagen-like protein CTD from *Legionella pneumophila* (PDB code 8QK8, chain A, orange). **E** β2 domain of ThomasA (gold) and mucin-binding domain from a cell surface protein of *Lactococcus lactis* (PDB code 7YL4, chain B, purple). **F-G** β5 domain of ThomasA (green) and Ig-like domains of GH5 cellulose hydrolase from *Bacillus licheniformis* (PDB code 4YZP, chain A, yellow) (**F**) and I10 domain of human titin (PDB code 5JDD, chain A, yellow) (**G**).

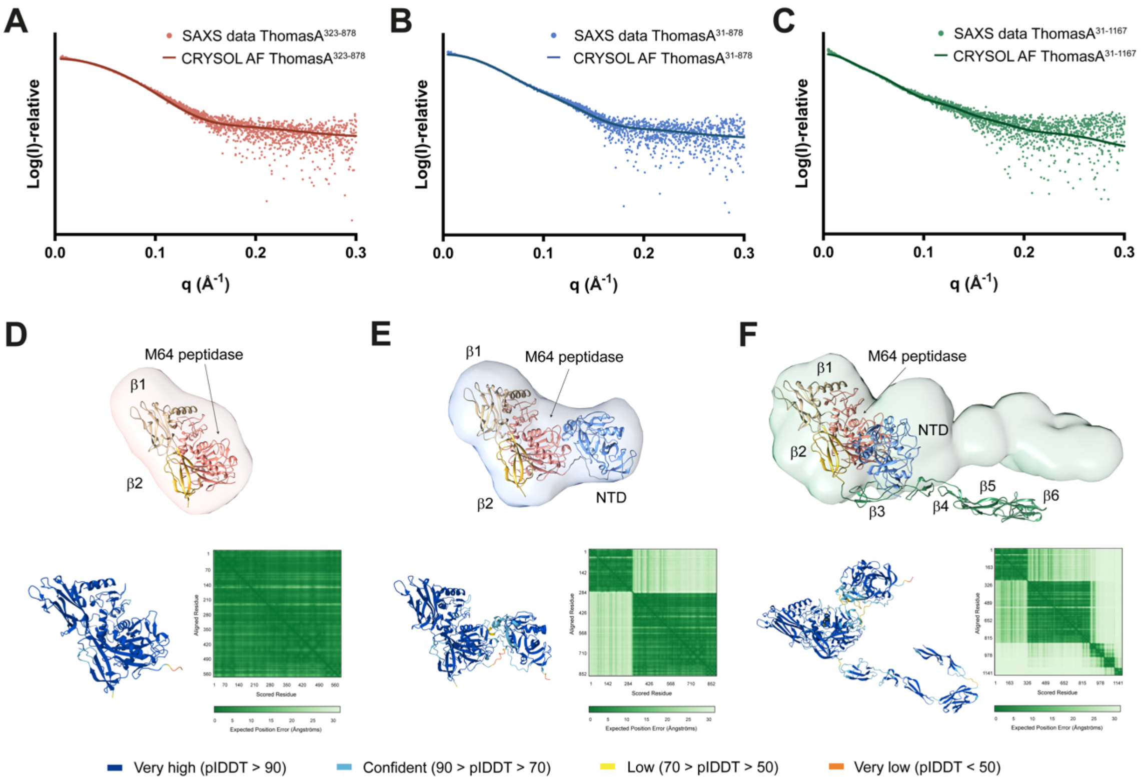

Appendix Figure S3. SAXS analysis of ThomasA constructs

A-**C** CRYSOL comparison of the solution scattering data (circles) to the theoretical scattering curve (line) of ThomasA$^{323\text{-}878}$ (χ^2 = 5.2) (**A**), ThomasA$^{31\text{-}878}$ (χ^2 = 4) (**B**) and ThomasA$^{31\text{-}1167}$ (χ^2 = 4.3) (**C**) AF3 models. **D**-**F** Most representative *ab initio* model volume of ThomasA$^{323\text{-}878}$ (χ^2 = 1.6) (**D**), ThomasA$^{31\text{-}878}$ (χ^2 = 2.1) (**E**) and ThomasA$^{31\text{-}1167}$ (χ^2 = 1.4) (**F**) using GASBOR. The AF3 models of ThomasA$^{323\text{-}878}$ (**D**), ThomasA$^{31\text{-}878}$ (**E**) and ThomasA$^{31\text{-}1167}$ (**F**) are shown in ribbons by the chimera's *fit in map* tool. In the lower panel, predicted aligned error (PAE) plots and per-residue confidence scores (pLDDT) are shown for the AF3 models of ThomasA$^{323\text{-}878}$ (**D**), ThomasA$^{31\text{-}878}$ (**E**), and ThomasA$^{31\text{-}1167}$ (**F**), illustrating the confidence in the relative positioning of residue pairs. High pLDDT values indicate high confidence in the local structure prediction, while low values may suggest flexible or disordered regions.

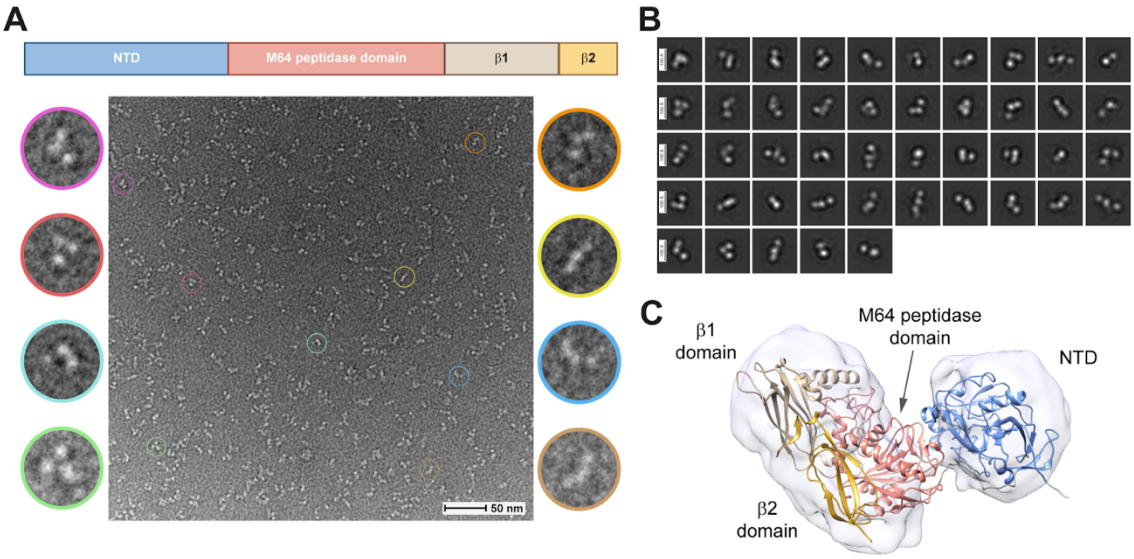

Appendix Figure S4. ThomasA$^{31\text{-}878}$ negative stain (NS) reconstruction.

A Micrograph detail of ThomasA$^{31\text{-}878}$. A small set of blurred and amplified particles has been chosen to show examples of picked particles (circle (scale bars, 100 Å). **C** 3D modeled reconstruction of *ab initio* estimation of ThomasA$^{31\text{-}878}$using selected 2D classes (surface) with superposition of AF3 model (ribbons).

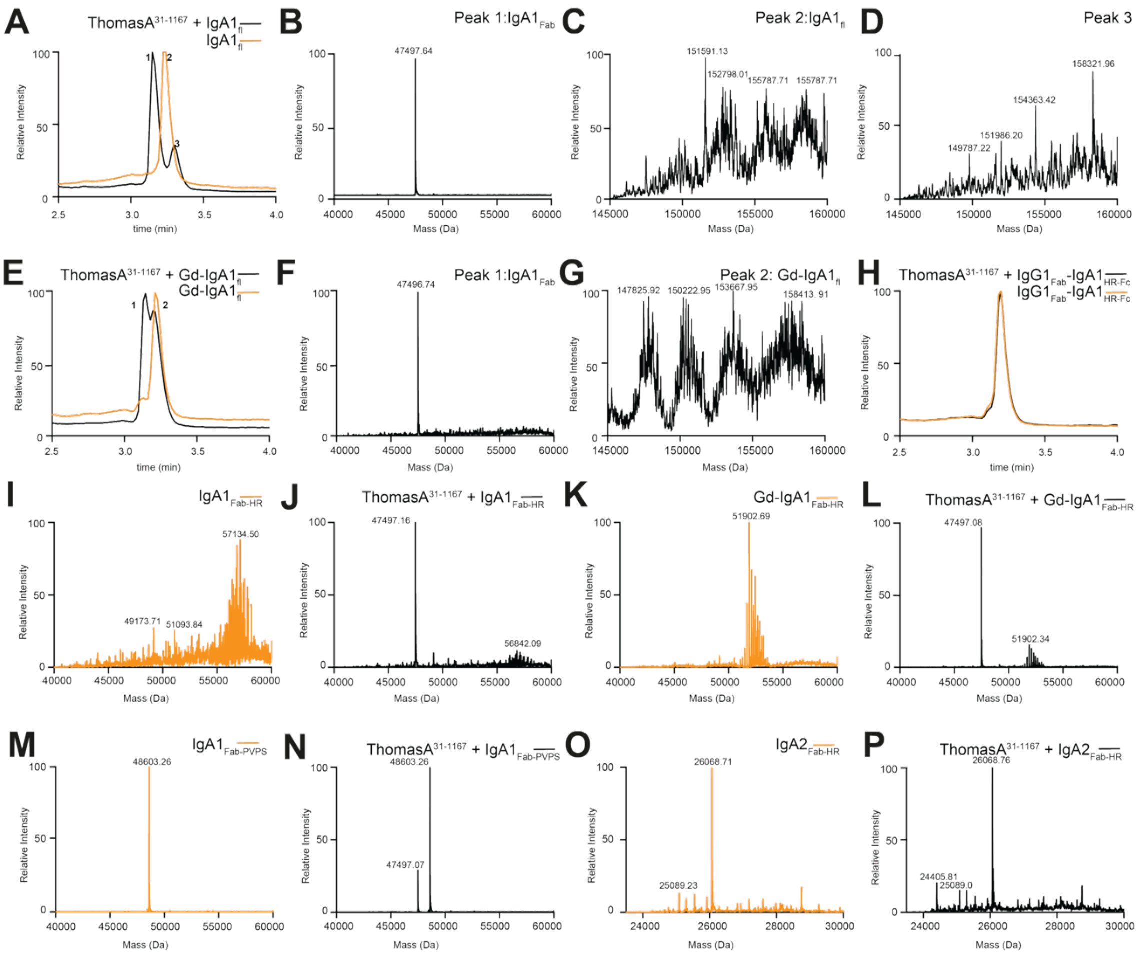

Appendix Figure S5. Intact LC-MS hydrolytic activity assays of ThomasA and IgA1 constructs.
A-H LC-MS analysis of IgA1$_{fl}$, Gd-IgA1$_{fl}$ and IgG1$_{Fab}$-IgA1$_{HR\text{-}Fc}$ reactions with ThomasA$^{31\text{-}1167}$. Chromatograms of the reaction with ThomasA$^{31\text{-}1167}$ and IgA1$_{fl}$ at t = 3 h (**A**), Gd-IgA1$_{fl}$ at t = 45 min (**E**) and IgG1$_{Fab}$-IgA1$_{HR\text{-}Fc}$ at t = 45 min (**H**). MS spectra depicting peak 1-3 (**B**-**D**) of reaction of ThomasA$^{31\text{-}1167}$ and IgA1$_{fl}$ at t = 3 h. Peak 1 is the IgA1$_{Fab}$ product of the reaction. MS spectra depicting peak 1-2 (**F**,**G**) of ThomasA$^{31\text{-}1167}$ and Gd-IgA1$_{fl}$ at t = 45 min. **I**-**P** MS spectra depicting the reactions with ThomasA$^{31\text{-}1167}$ and IgA1$_{Fab\text{-}HR}$ at t = 0 min (**I**), IgA1$_{Fab\text{-}HR}$ at t = 45 min (**J**), Gd-IgA1$_{Fab\text{-}HR}$ at t = 0 min (**K**), Gd-IgA1$_{Fab\text{-}HR}$ at t = 45 min (**L**), IgA1$_{Fab\text{-}PVPS}$ at t = 0 min (**M**), IgA1$_{Fab\text{-}PVPS}$ at t = 45 min (**N**), IgA2$_{Fab\text{-}HR}$ at t = 0 min (**O**) and IgA2$_{Fab\text{-}HR}$ at t = 45 min (**P**).

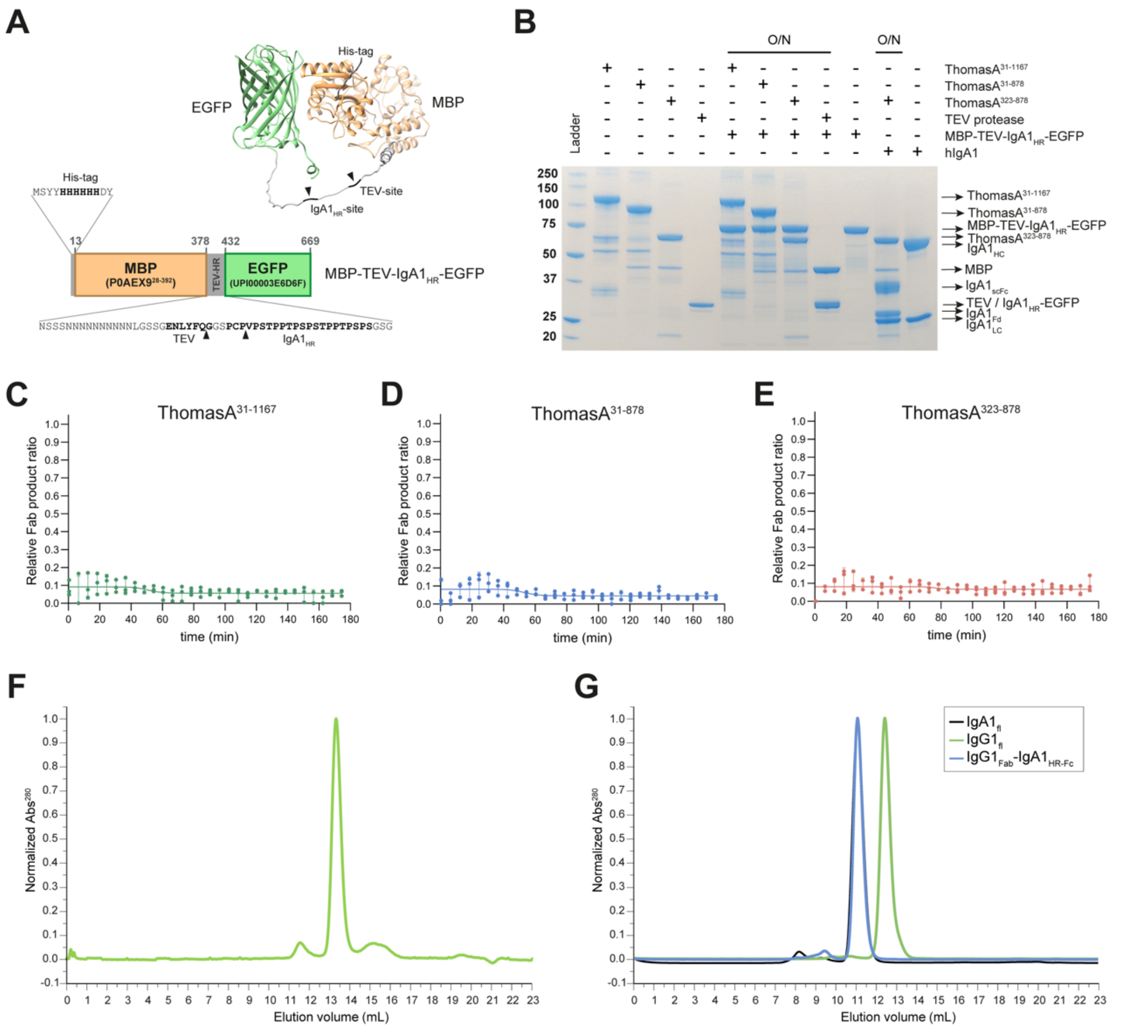

Appendix Figure S6. Hydrolytic activity of ThomasA constructs against MBP-TEV-IgA1$_{HR}$-EGFP and IgG1$_{Fab}$-IgA1$_{HR-Fc}$.

A Schematic representation and AF3 model of the MBP-TEV-IgA1$_{HR}$-EGFP construct. **B** SDS-PAGE analysis of the hydrolytic activity of ThomasA$^{31-1167}$, ThomasA$^{31-878}$, ThomasA$^{323-878}$ and TEV protease against MBP-TEV-IgA1$_{HR}$-EGFP. **C-E** LC-MS analysis of hydrolytic activity of ThomasA$^{31-1167}$ (**C**), ThomasA$^{31-878}$ (**D**) and ThomasA$^{323-878}$ (**E**) against IgG1$_{Fab}$-IgA1$_{HR-Fc}$. **F-G** Size exclusion chromatography profiles of MBP-TEV-IgA1$_{HR}$-EGFP (**F**) and IgG1$_{Fab}$-IgA1$_{HR-Fc}$ (**G**).

A

GGSGPDWNVSTEQSIQLTLGPWYSNDGKYSNPTIPVYTIQKTRSDTENMVVVVCGEGYTKSQQGKFINDVKRLWQDAMKY
EPYRSYADRFNVYALCTASESTFDNGGSTFFDVIVDKYNSPVISNNLHGSQWKNHIFERCIGPEFIEKIHDAHIKKKCDP
NTIPSGSEYEPYYYVHDYIAQFAMVVNTKSDFGGAYNNREYGFHYFISPSDSYRASKTFAHEFGHGLLGLGDEYSNGYLL
DDKELKSLNLSSVEDPEKIKWRQLLGFRNTYTCRNAYGSKMLVSSYECIMRDTNYQFCEVCRLQGFKRMSQLVKDVDLYV
ATPEVKEYTGAYSKPSDFTDLETSSYYNYTYNRNDRLLSGNSKSRFNTNMNGKKIELRTVIQNISDKNARQLKFKMWIKH
SDGSVATDSSGNPLQTVQTFDIPVWNDKANFWPLGALDHIKSDFNSGLKSCSLIYQIPSDAQLKSGDTVAFQVLDENGNV
LADDNTETQRYTTVSIQYKFEDGSEIPNTAGGTFTVPYGTKLDLTPAKTLYDYEFIKVDGLNKPIVSDGTVVTYYYKNKN

B

GAQNFSDYFTNKTLRIDYLFTGNADKQSICLDELSELPVWAGRRHHLSELPLEGNGQIVMRDVASGKVIYTTSFSSLFQE
WLETDEAKEVTKGFENTYLLPYPIKPAEVEITLRNNKREVSANLKHVVKPDDILIHKKGLTHITPHKYLLKSGNEEQCID
VAILAEGYTTSEMETFYKDAAIACEALFSHEPFQSMKNRFNIVAVASPSADSGVSAPKQGAWKHTAFGSHFDTFYSDRYL
TTSRVKAINDALAGIPYEHIIILANTEQYGGGGIYNAFTLTTAHHPNFRPVVVHEFGHSFGGLADEYFYDEDVMNGLYPL
NIEPWEQNITTRINFASKWEDMLTKTTPVPTPVADKAKYPIGVYEGGGYSAKGIYRPAFDCRMRTNEYPTFCPVCQRAIQ
RIIEFYTGK

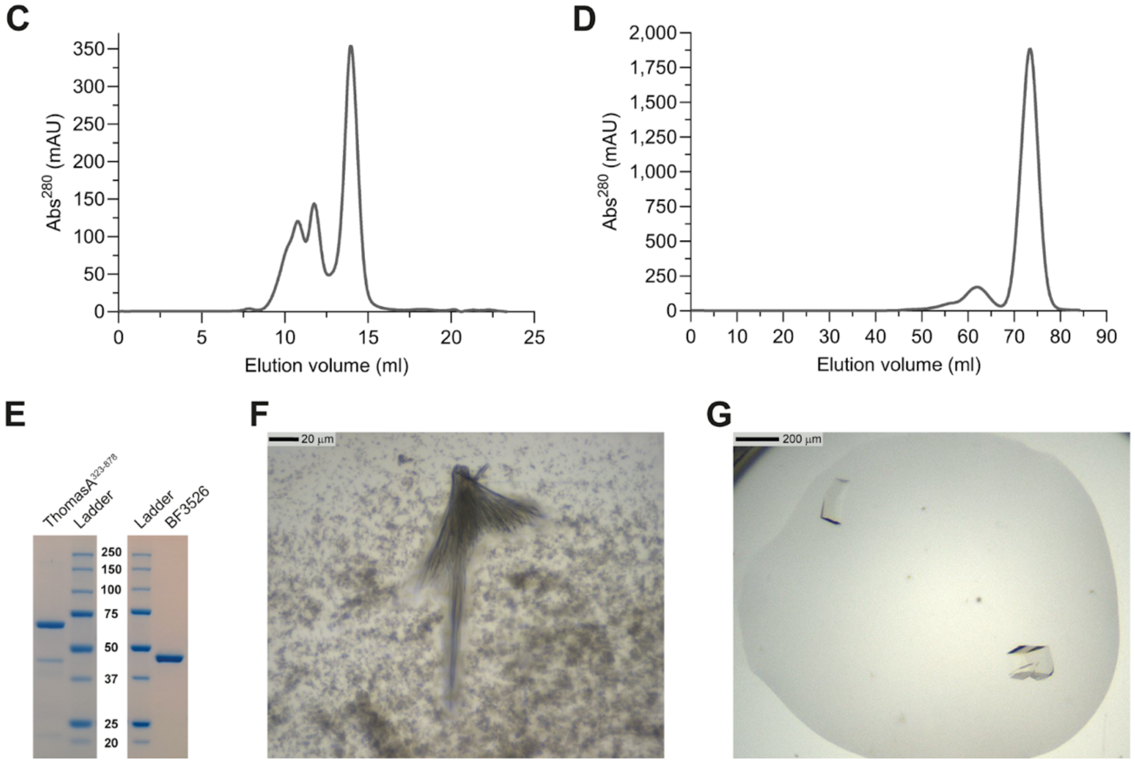

Appendix Figure S7. Recombinant production of ThomasA$^{323\text{-}878}$ and BF3526.

A,B Sequences of recombinant constructs of ThomasA$^{323\text{-}878}$ (**A**) and BF3526 (**B**) after TEV protease cleavage. **C,D** Gel filtration profile of ThomasA$^{323\text{-}878}$ (**C**) and BF3526 (**D**) using the Superdex 200 columns HiLoad 10/300GL and HiLoad 16/600, respectively. **E** SDS-PAGE of pure ThomasA$^{323\text{-}878}$ and BF3526 used for X-ray crystallography. **F,G** Pictures of crystals of ThomasA$^{323\text{-}878}$ rods (**F**) and BF3526 cubes (**G**) used to get data collection of X-ray structures (PDB codes 9QDH and 9QDI).

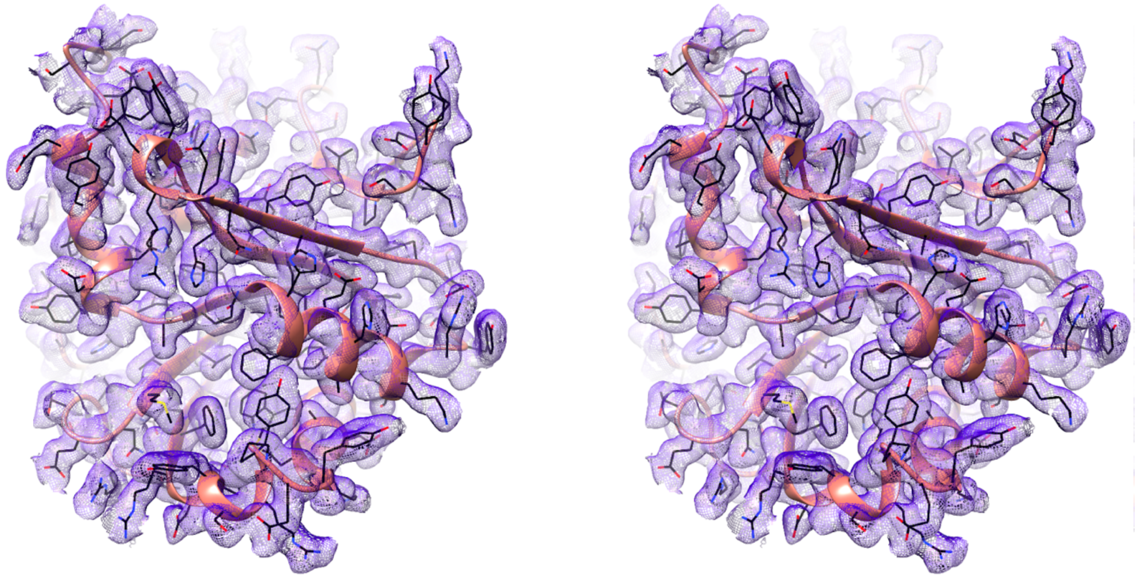

Appendix Figure S8. Electron density maps of the refined ThomasA$^{323\text{-}878}$ X-ray crystal structure.

Stereo views of the final electron density map (2mF$_o$ –DF$_c$ contoured at 1σ) corresponding to the ThomasA$^{328\text{-}878}$ apo structure.

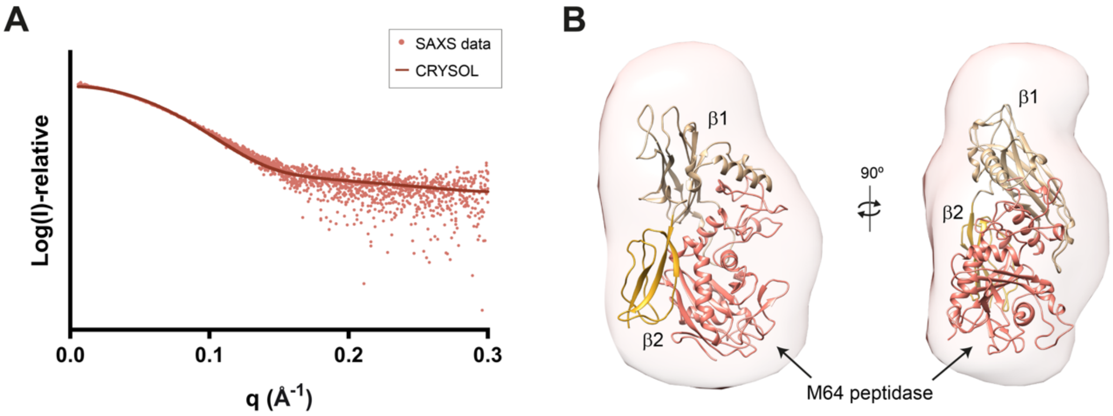

Appendix Figure S9. SAXS analysis of ThomasA$^{323\text{-}878}$.

A CRYSOL comparison of the solution scattering data (circles) to the theoretical scattering curve (line) of ThomasA$^{323\text{-}878}$ X-ray crystal structure ($\chi^2 = 5.460$). **B** Most representative *ab initio* model of ThomasA$^{323\text{-}878}$ using GASBOR ($\chi^2 = 1.602$) in two different orientations. The crystal structure of ThomasA$^{323\text{-}878}$ is shown in ribbons by the chimera's *fit in map* tool.

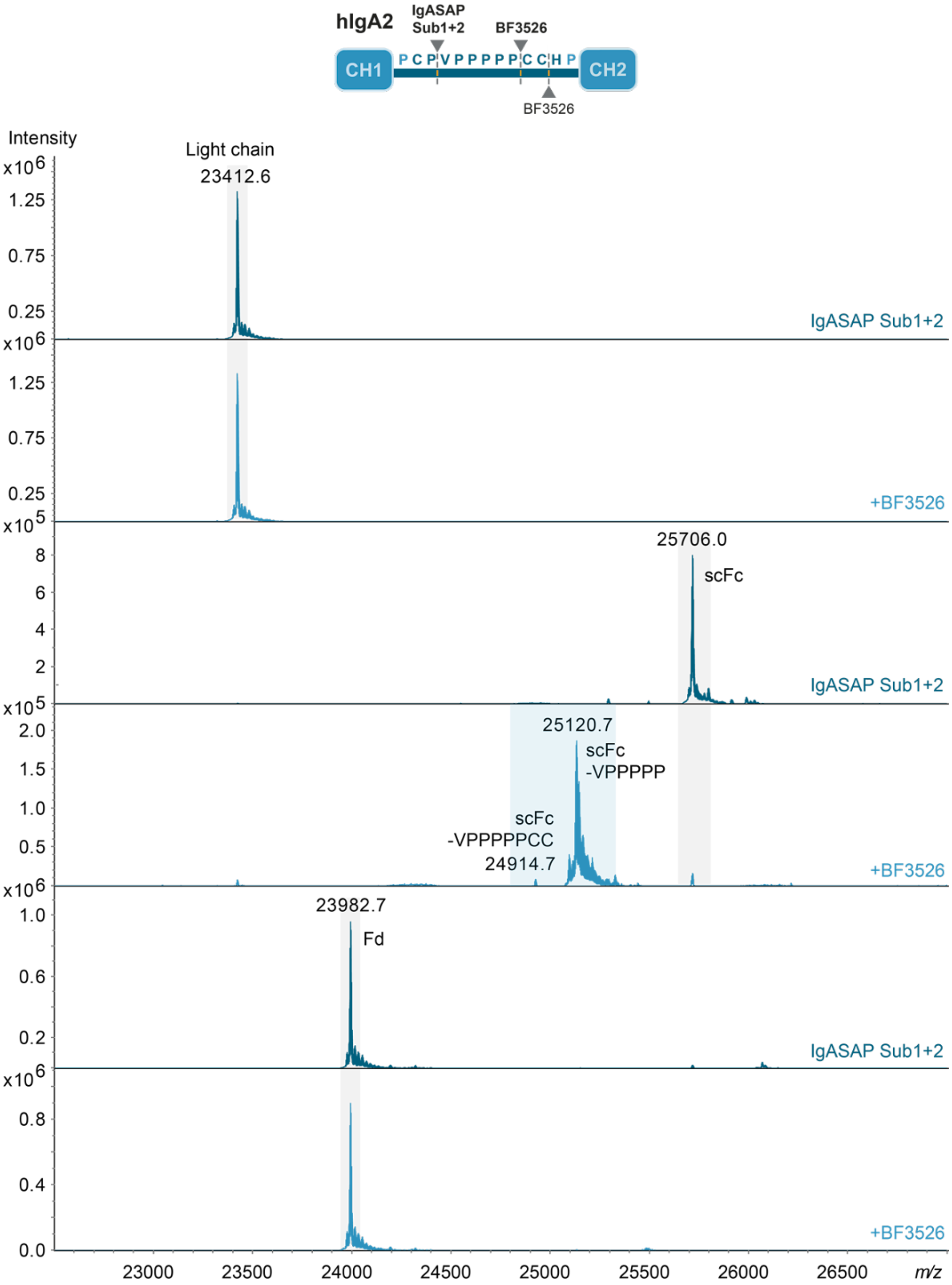

Appendix Figure S10. Hydrolytic activity of BF3526 towards predigested hIgA2 by IgASAP Sub1+2.

Deconvoluted mass spectra of subunits of hIgA2 generated after IgASAP Sub1+2 digestion (dark blue) or IgASAP Sub1+2 and BF3526 digestion (light blue).

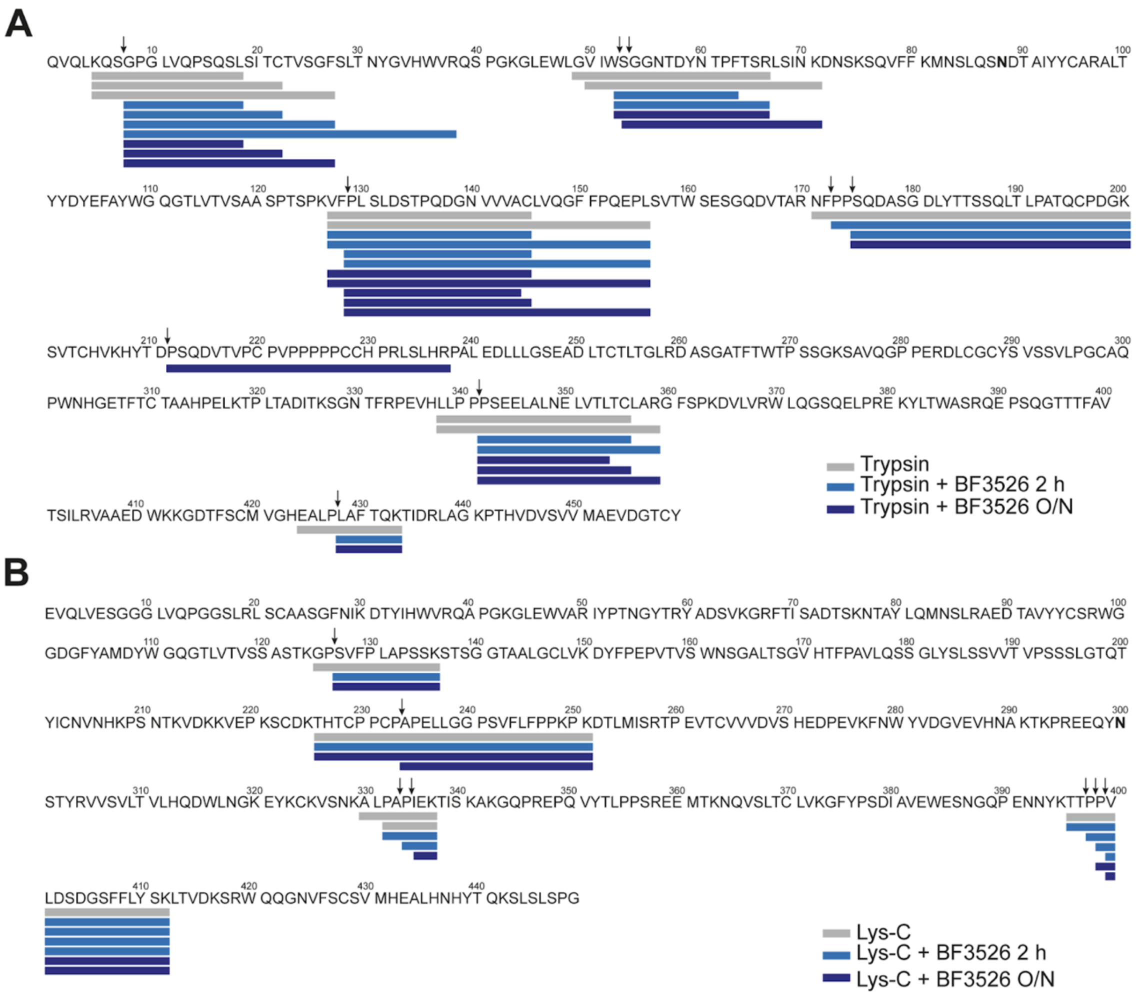

Appendix Figure S11. Hydrolytic activity of BF3526 towards hIgA2 and IgG1 peptides analyzed by LC-MS/MS.

A Peptide mapping of trypsin-digested hIgA2 with or without BF3526 digestion. **B** Peptide mapping of Lys-C-digested IgG1 with or without BF3526 digestion.

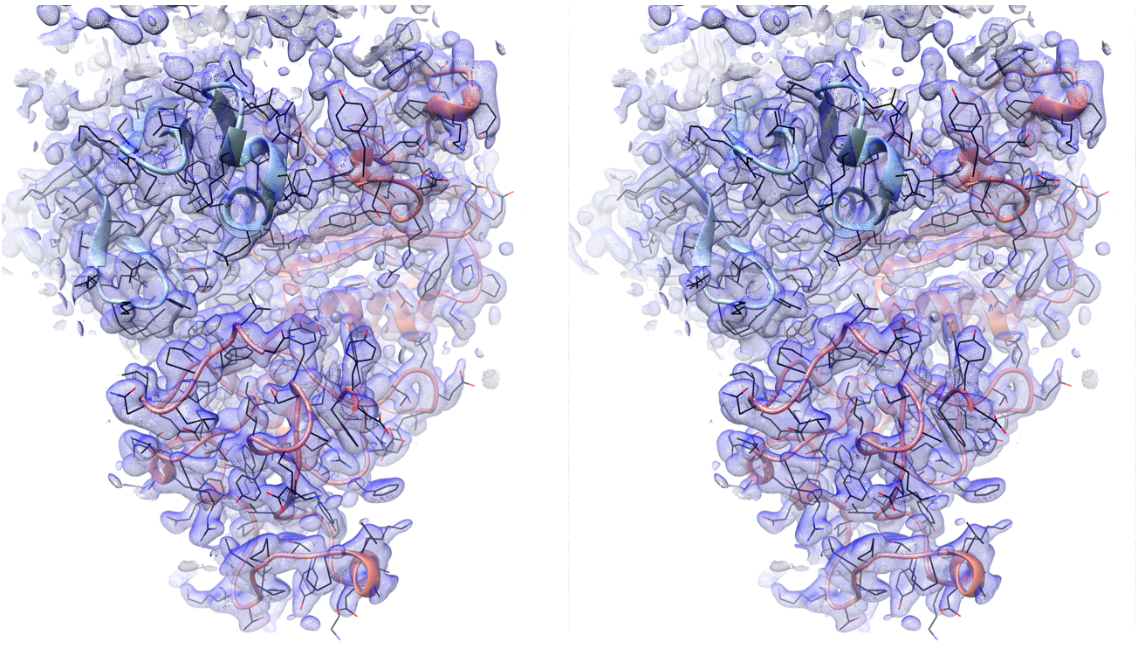

Appendix Figure S12. Electron density maps of the refined BF3526 X-ray crystal structure. Stereo view of the final electron density maps ($2mF_o–DF_c$ contoured at 1σ) corresponding to the BF3526-unliganded structure.

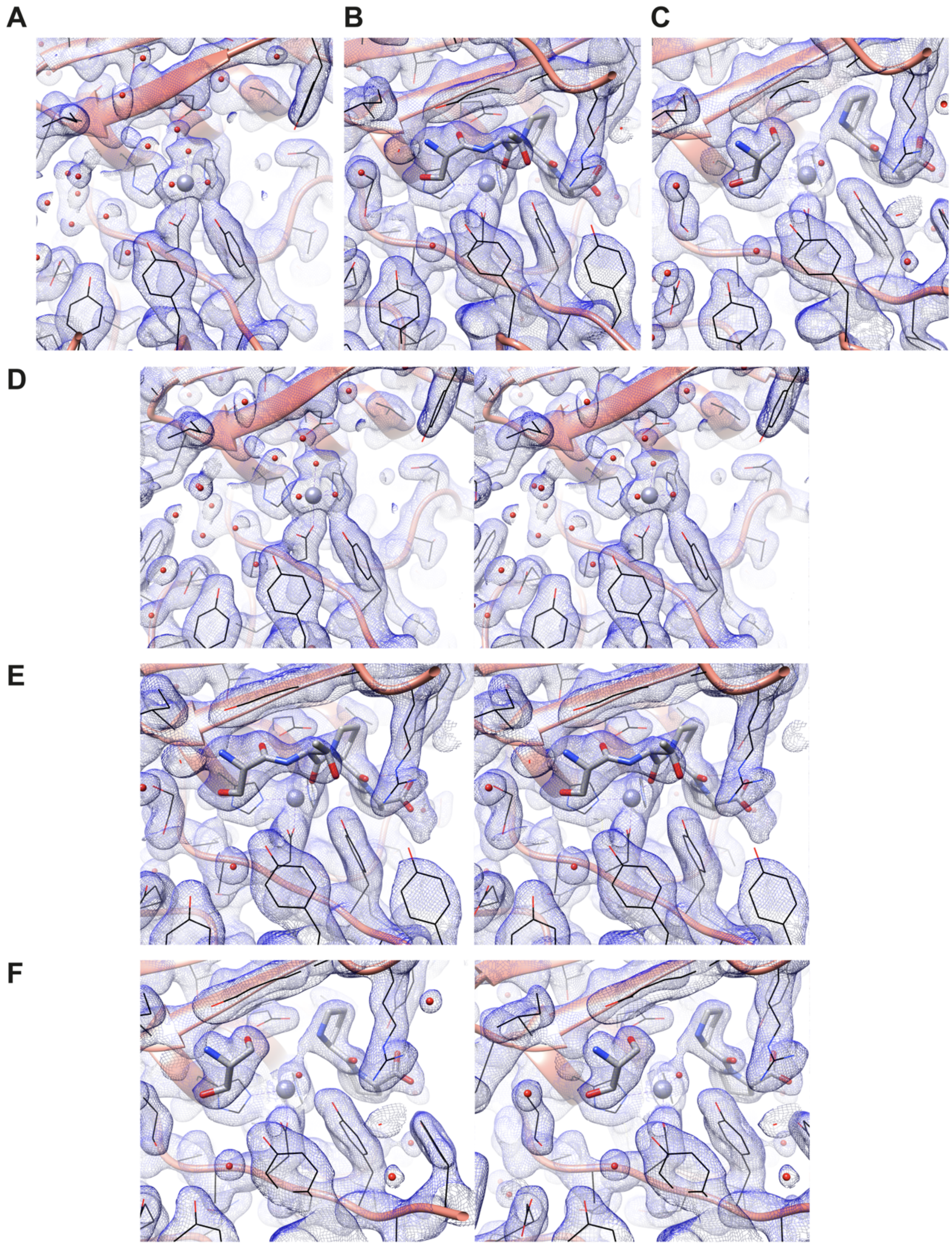

Appendix Figure S13. Electron density maps of the active site of BF3526-unliganded, BF3526-STPP and BF3526-PP molecules.

A-C. Standard orientation views of the electron density maps of the active site of BF3526-unliganded (**A**), BF3526-STPP (**B**) and BF3526-PP (**C**) molecules. **D-F** Stereo views of the electron density maps corresponding to the BF3526-unliganded (**D**), BF3526-STPP (**E**) and BF3526-PP (**F**) molecules. All maps show $2mF_o-DF_c$ electron density contoured at 1σ.

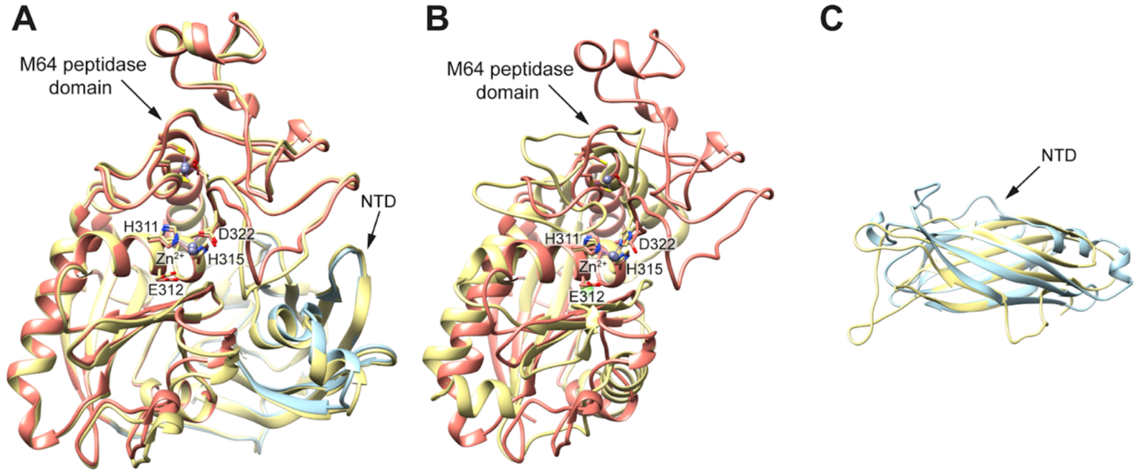

Appendix Figure S14. Structural homologues of BF3526 domains.

A-C Structural superposition of the X-ray crystal structure of BF3526 (M64 peptidase domain in salmon and NTD in light blue; chain C) with M64 peptidase family BACOVA_00663 from *B. ovatus* (PDB code 3P1V, chain A, yellow) (**A**), snake venom metalloproteinase M12 metalloproteinase leucurolysin-a from *B. leucurus* (PDB code 4Q1L, chain A, yellow) (**B**) and a carbohydrate-binding domain of the beta-glucuronidase from *B. uniformis* (PDB code 5UJ6, chian B, yellow) (**C**).

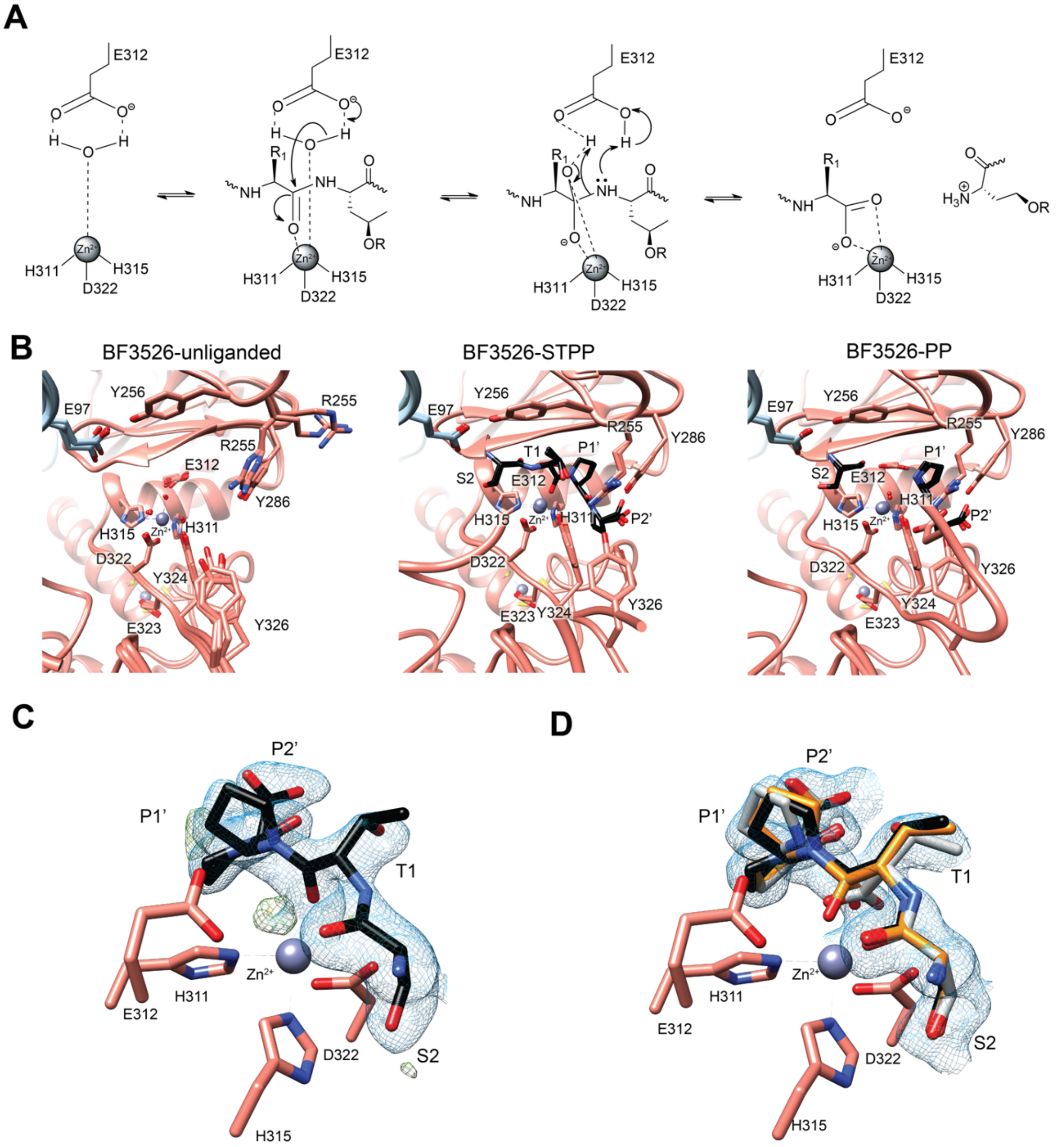

Appendix Figure S15. Catalytic mechanism of BF3526.

A Proposed catalytic mechanism for BF3526. **B** Catalytic site of the superimposed BF3526-unliganded molecules (left), BF3526-STPP (center) and BF3526-PP (right) molecules in the BF3526 crystal structure. **C** Final electron density maps ($2mF_o–DF_c$ contoured at 1σ (blue) and $mF_o–DF_c$ at 1σ (green)) corresponding to the STPP substrate in the active site. Residues involved in catalysis in the BF3526-STTP structure are highlighted. **D** Featured Enhanced Map (FEM) obtained by removing the STPP peptide from the active site. Superposition of the STPP substrate (black), ST-PP product (grey) and the *gem*-diolate intermediate (orange) locally refined in Coot. Residues involved in catalysis in the BF3526-STTP structure are highlighted.

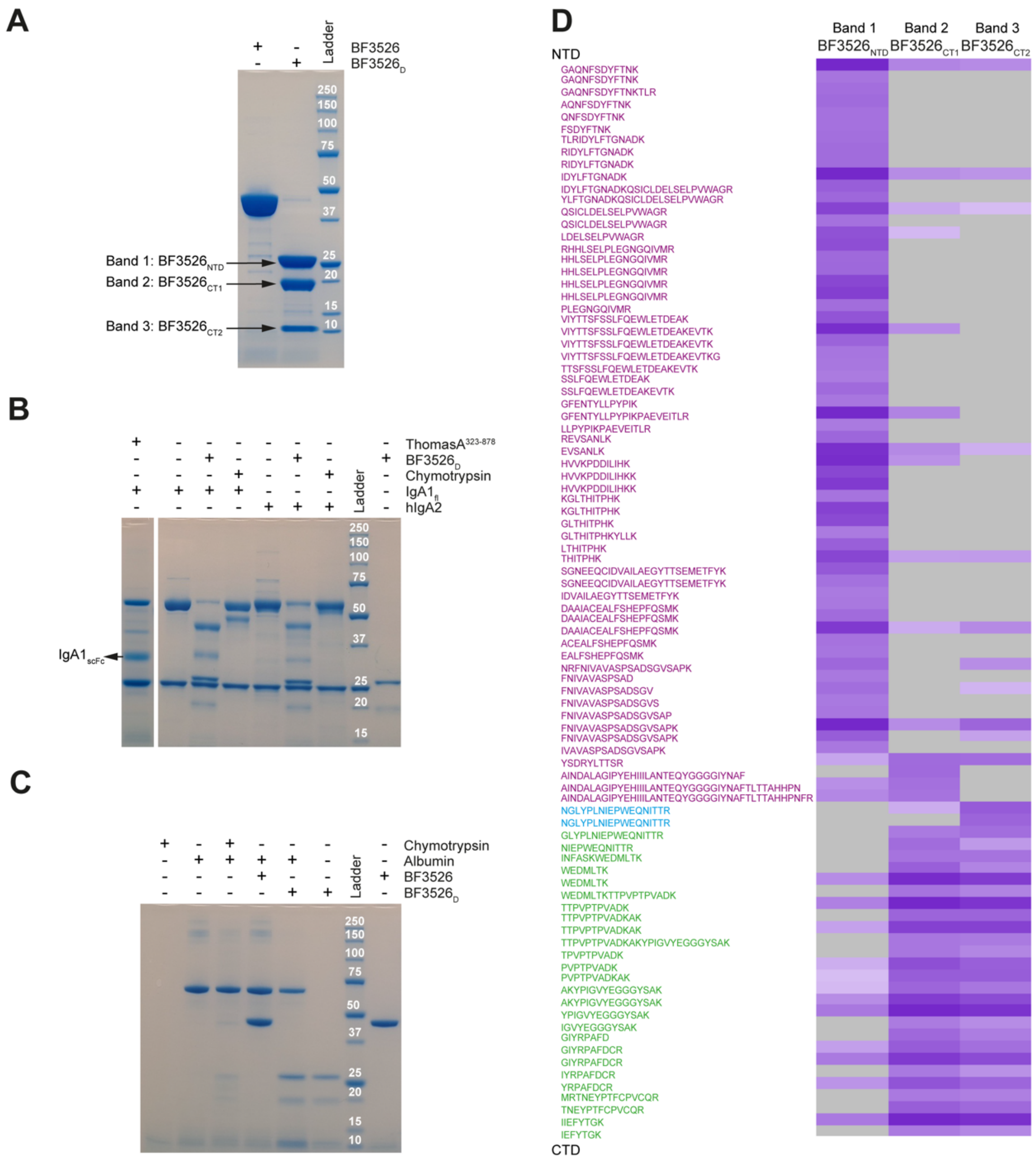

Appendix Figure S16. Hydrolytic activity of $BF3526_{NTD}$, $BF3526_{CT1}$ and $BF3526_{CT2}$ against $IgA1_{fl}$, hIgA2 and IgG1.

A SDS-PAGE showing the three bands corresponding to BF3526 after digestion with chymotrypsin ($BF3526_{D}$). **B** Hydrolytic activity of $BF3526_{NTD}$, $BF3526_{CT1}$ and $BF3526_{CT2}$ against $IgA1_{fl}$ and hIgA2. **C** Hydrolytic activity of $BF3526_{NTD}$, $BF3526_{CT1}$ and $BF3526_{CT2}$ against albumin. **D** Peptide heat map of the LC-MS analysis of $BF3526_{D}$ SDS-PAGE bands.

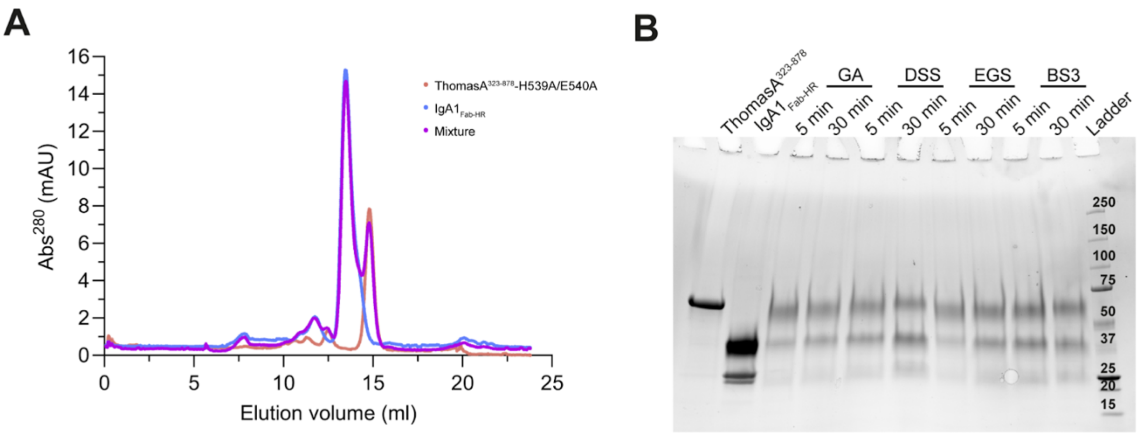

Appendix Figure S17 | Analysis by size exclusion and SDS-PAGE of ThomasA$^{323\text{-}878}$ and IgA1$_{Fab\text{-}HR}$ complex formation.

A Size exclusion chromatography of ThomasA$^{323\text{-}878}$-H539A/E540A (mixture; salmon), IgA1 $_{Fab\text{-}HR}$ (blue) and mixture of both proteins in a 1:5 ratio (purple), using the Superdex 200 column HiLoad 10/300GL. **B** SDS-PAGE analysis of the cross-linking complex formation of ThomasA$^{323\text{-}878}$ and IgA1$_{Fab\text{-}HR}$ in a 1:5 ratio using glutaraldehyde (GA), disuccinimidyl suberate (DSS), ethylene glycol bis(succinimidyl succinate) (EGS) and bis(sulfosuccinimidyl)syberate (BS3).

3. Appendix text

Structural homologues of ThomasA individual domains

A structural homology search using the DALI server (Holm *et al*, 2023) identified peptidases with significant structural similarity to the M64 peptidase domain of ThomasA. The closest homologs include the M64 peptidase from *Bacteroides fragilis* (*strain NCTC 9343*), BF3526 (PDB code 4DF9; Z-score of 25.5; root mean squared deviation (r.m.s.d.) of 2.3 Å for 234 aligned residues; 21% identity), the M64 peptidase from *B. ovatus* BACOVA_00663 (PDB code 3P1V; Z-score of 22.7, r.m.s.d. of 2.5 Å for 233 aligned residues; 22% identity), and the M12 peptidase leucurolysin-a from *Bothrops leucurus* snake venom (PDB code 4Q1L; Z-score of 10.4; r.m.s.d. of 3.2 Å for 156 aligned residues; 21% identity), all of which belong to the metzincin clan (Cerdà-Costa & Gomis-Rüth, 2014) (Appendix Fig S2A-C). Notably, BF3526 and BACOVA_00663 belong to cluster CL-I in our SSN analysis but have not yet been biochemically characterized. In contrast, the NTD, β2, and β5-sandwich domains exhibited low structural similarity to known proteins, while the remaining domains lacked sufficient similarity to reported structures in the PDB database (Appendix Fig S2D,G). The closest structural homolog of the NTD is DUF1566 of a collagen-like protein from *Legionella pneumophila* strain 130b (Rehman *et al*, 2023) (PDB code 8QK8; Z-score of 7.4; r.m.s.d. of 4 Å for 155 aligned residues; 13% identity) (Appendix Fig S2D). The β2-sandwich domain shares structural homology with a mucin-binding protein domain from *Lactococcus lactis* (PDB code 7YL4; Z-score of 7.7; r.m.s.d. of 2.6 Å for 74 aligned residues; 21% identity) (Appendix Fig S2E). The β5-sandwich domain displays low structural homology to Ig-like domain structures, with two representative examples. The first is an Ig-like domain from a GH5 cellulose hydrolase of *Bacillus licheniformis*, which functions as an auxiliary domain (Liberato *et al*, 2016) (PDB code 4YZP; Z-score of 10.1; r.m.s.d. of 2.7 Å for 87 aligned residues; 21% identity) (Appendix Fig S2F). The second is human titin, a structural intrasarcomeric protein of striated muscle (Bogomolovas *et al*, 2016) (PDB code 5JDD; Z-score of 9.1; r.m.s.d. of 2.2 Å for 79 aligned residues; 19% identity) (Appendix Fig S2G).

Structural homologues of BF3526

A structural homology search using the DALI server (Holm *et al*, 2023) revealed only one protein with high structural homology to BF3526, BACOVA_00663 from *B. ovatus* ATCC 8483 (PDB code 3P1V; Z-score of 66.2; r.m.s.d. of 0.9 Å for 405 aligned residues; 73% identity; Appendix Fig S14A). This protein is also classified in the M64 family; however, the substrate specificity and function are unknown. The M64 peptidase domain also shows low structural homology with leucurolysin-a, a M12 metalloprotease from *B. leucurus* (PDB code 4Q1L; Z-score of 10.6; r.m.s.d. of 3.2 Å for 155 aligned residues; 19% identity; Appendix Fig S14B). The NTD of BF3526 shows low structural homology with one of the β-sandwich domains of the beta-glucuronidase from *B. uniformis* str. 3978 T3 ii (Pollet *et al*, 2017) (PDB code 5UJ6; Z-score of 7.3; r.m.s.d. of 2.5 Å for 83 aligned residues; 19% identity; Appendix Fig S14C).

4. References

Bogomolovas J, Fleming JR, Anderson BR, Williams R, Lange S, Simon B, Khan MM, Rudolf R, Franke B, Bullard B, *et al* (2016) Exploration of pathomechanisms triggered by a single-nucleotide polymorphism in titin's I-band: the cardiomyopathy-linked mutation T2580I. *Open Biol* 6: 160114

Cerdà-Costa N & Gomis-Rüth FX (2014) Architecture and function of metallopeptidase catalytic domains. *Protein Science* 23: 123–144

Holm L, Laiho A, Törönen P & Salgado M (2023) DALI shines a light on remote homologs: One hundred discoveries. *Protein Science* 32: e4519

Liberato MV, Silveira RL, Prates ÉT, De Araujo EA, Pellegrini VOA, Camilo CM, Kadowaki MA, Neto MDO, Popov A, Skaf MS, *et al* (2016) Molecular characterization of a family 5 glycoside hydrolase suggests an induced-fit enzymatic mechanism. *Sci Rep* 6: 23473

Rehman S, Antonovic AK, McIntire IE, Zheng H, Cleaver L, Adams CO, Portlock T, Richardson K, Shaw R, Oregioni A, *et al* (2023) The *Legionella* collagen-like protein employs a unique binding mechanism for the recognition of host glycosaminoglycans. doi:10.1101/2023.12.10.570962 [PREPRINT]
