# Supplementary figures and images for: Molecular basis of Fab-dependent IgA antibody recognition by gut-bacterial metallopeptidases
Source: EMBO J. 2025 Jul 31;44(17):4867–98. doi: 10.1038/s44318-025-00518-w (PMC12402451; doi:10.1038/s44318-025-00518-w)

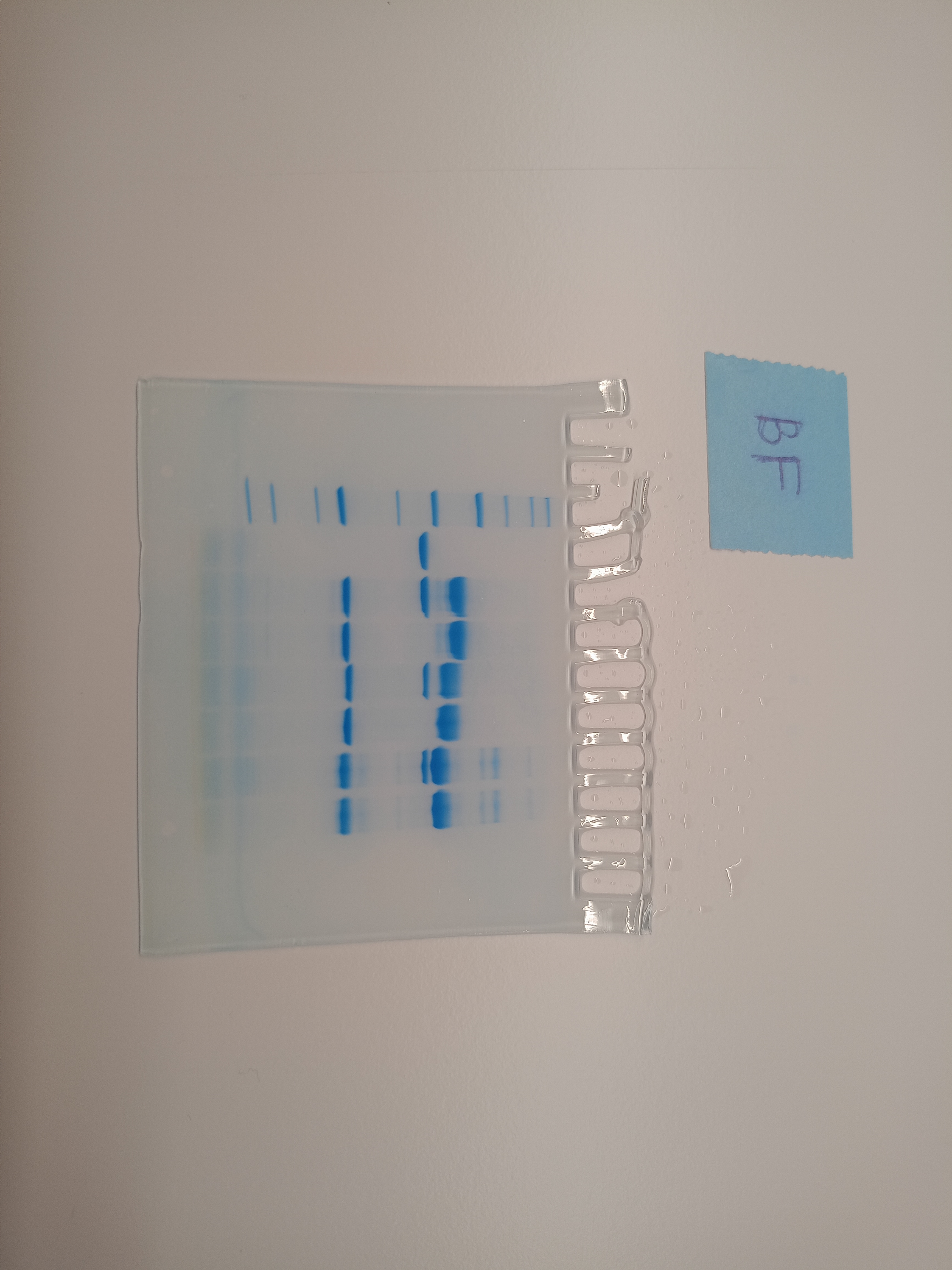

Supplement: Supplementary file 6 — Source data Fig. 5 [file 44318_2025_518_MOESM6_ESM.zip › EMBOJ-2025-120971_Fig5/5A/EMBOJ-2025-120971_ImageFig5A.jpg]

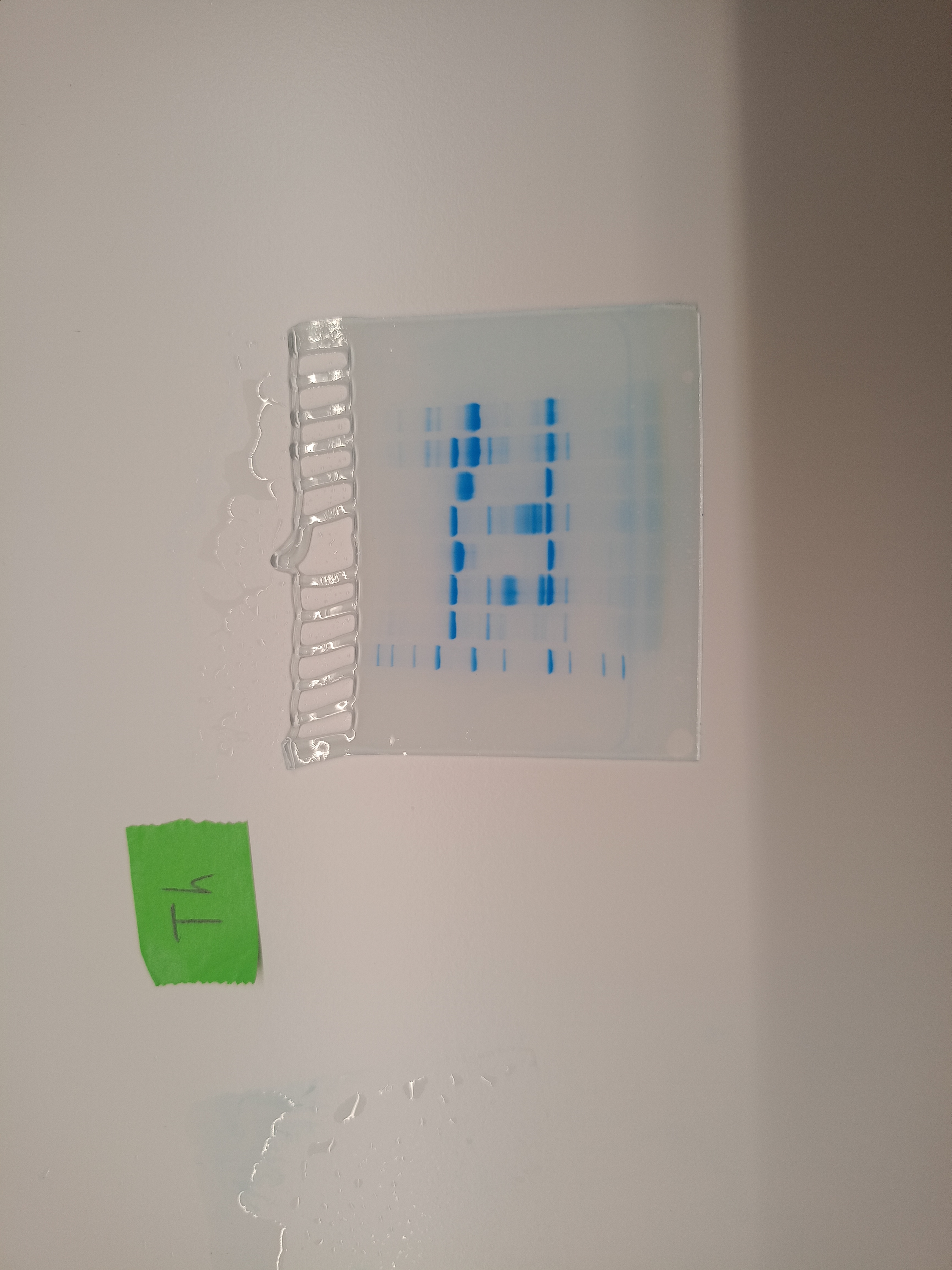

Supplement: Supplementary file 6 — Source data Fig. 5 [file 44318_2025_518_MOESM6_ESM.zip › EMBOJ-2025-120971_Fig5/5B/EMBOJ-2025-120971_ImageFig5B.jpg]
