# Supplementary material for: Molecular basis of Fab-dependent IgA antibody recognition by gut-bacterial metallopeptidases
Source: EMBO J. 2025 Jul 31;44(17):4867–98. doi: 10.1038/s44318-025-00518-w (PMC12402451; doi:10.1038/s44318-025-00518-w)
Supplement: Supplementary file 9 — Expanded View Figures [file 44318_2025_518_MOESM9_ESM.pdf]

Expanded View Figures

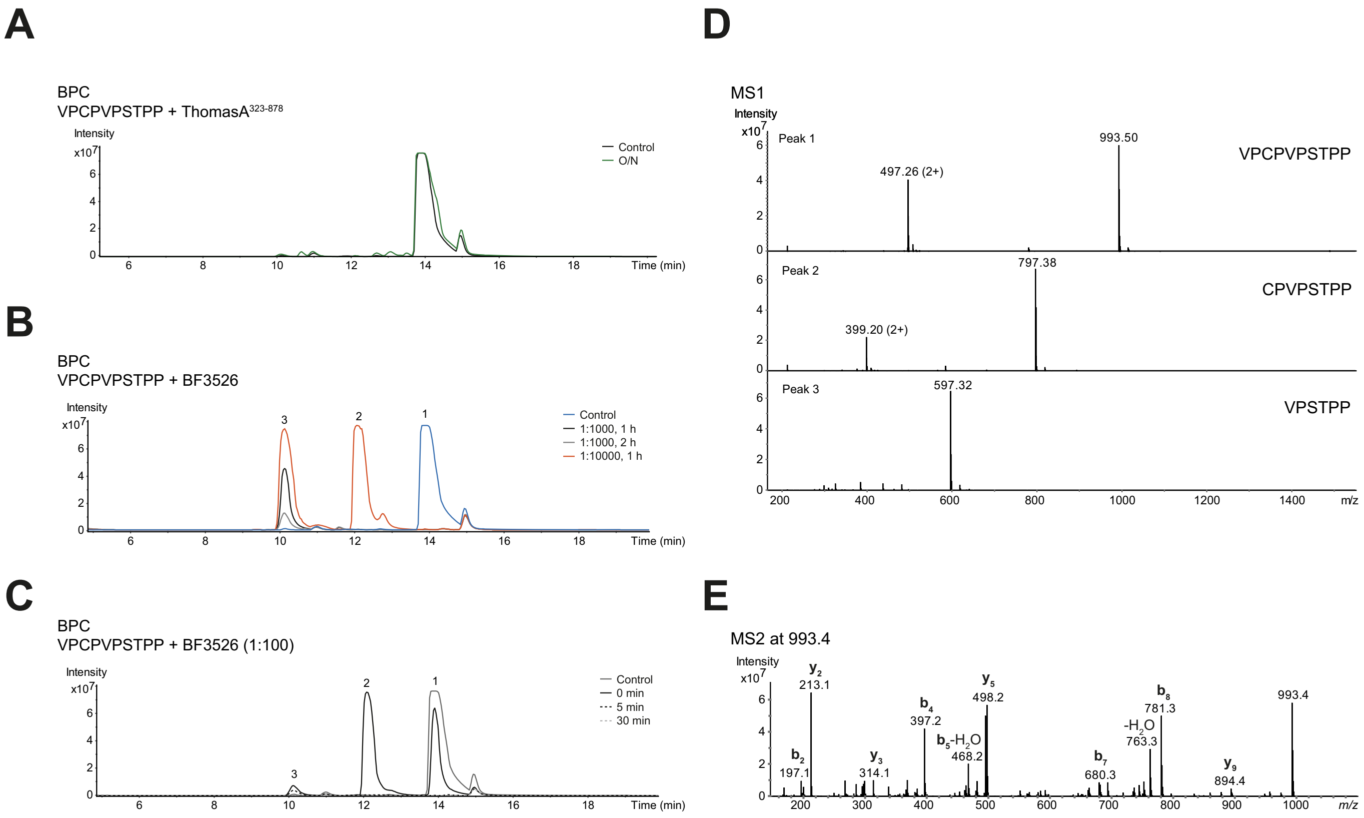

Figure EV1. Hydrolytic activity of BF3526 and ThomasA$^{323-878}$ against the decapeptide VPCPVPSTPP.

(**A–C**) Base peak chromatograms (BPCs) of the reaction of ThomasA$^{323-878}$ (**A**) and BF3526 (**B**, **C**) with the decapeptide VPCPVPSTPP. (**D**) MS1 spectra of Peak 1-3. (**E**) MS2 at *m/z* 993.4, which corresponds to the intact VPCPVPSTPP peptide. All enzymatic activity measurements were performed in triplicate.

© The Author(s)

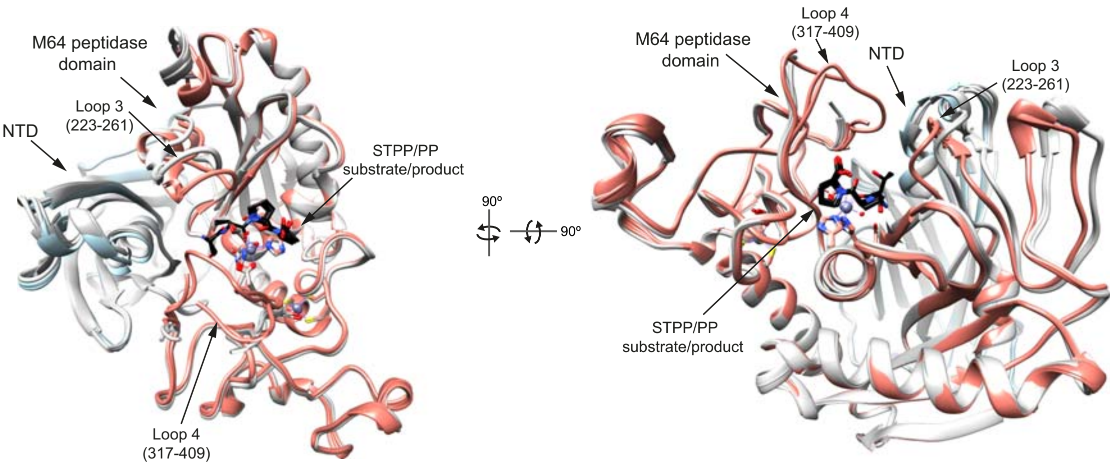

Figure EV2. Structural comparison of BF3526-unliganded, BF3526-PP and BF3526-STPP molecules.

Two views of the superposition of BF3526-unliganded (shown in gray) with BF3526-PP (shown in light blue and salmon) and BF3526-STPP (shown in light blue and salmon).

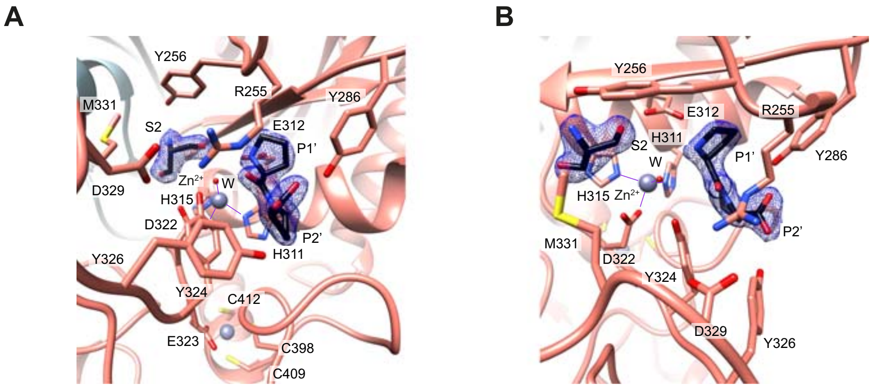

Figure EV3. Active site of BF3526-PP.

(**A**, **B**) Two views of the BF3526-STPP structure in cartoon representation, showing the location of S2T1P1′P2′ substrate within the active site and the key interacting residues. The electron density map of the S2 and P1′P2′ product is shown at 1.0 σ r.m.s.d.

 © The Author(s)

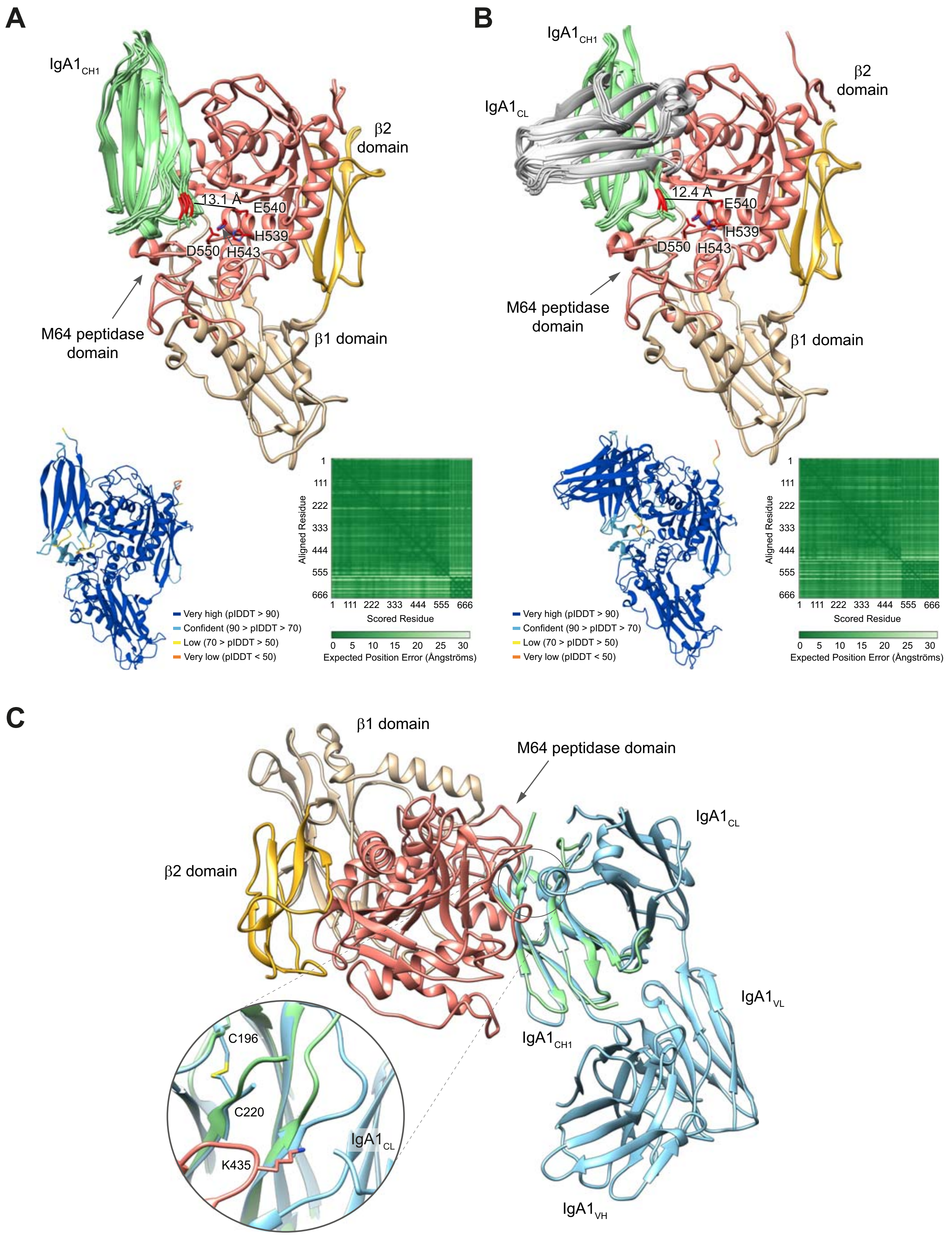
A
IgA1CH1
β2 domain
13.1 Å
E540
H539
D550
H543
M64 peptidase domain
β1 domain
Very high (pIDDT > 90)
Confident (90 > pIDDT > 70)
Low (70 > pIDDT > 50)
Very low (pIDDT < 50)
Aligned Residue
Scored Residue
Expected Position Error (Ångströms)
B
IgA1CH1
IgA1CL
β2 domain
12.4 Å
E540
H539
D550
H543
M64 peptidase domain
β1 domain
C
β1 domain
M64 peptidase domain
IgA1CL
β2 domain
IgA1VL
IgA1CH1
C196
C220
IgA1CL
K435
IgA1VH

Figure EV4. AF3 model analysis of ThomasA$^{323\text{-}878}$-IgA1$_{CH1}$ and ThomasA$^{323\text{-}878}$-IgA1$_{CH1\text{-}CL}$ complexes formation.

(**A, B**) Superposition of five ThomasA$^{323\text{-}878}$-IgA1$_{CH1}$ (**A**) and ThomasA$^{323\text{-}878}$-IgA1$_{CH1\text{-}CL}$ (**B**) complex models predicted by AF3. M64 peptidase domain, β1, and β2, CH1 and CL domains are colored in salmon, beige, and gold, green and gray, respectively (upper panels). Predicted aligned error (PAE) plots and per-residue confidence scores (pLDDT) for the AF3 complex models are shown in the lower panels. (**C**) Superposition of IgA1$_{Fab}$ structure (PDB code 7K75, chains A and B, blue) and the AF3 ThomasA$^{323\text{-}878}$-IgA1$_{CH1}$ complex model. A close-up view highlights the C196-C220 cysteine bond and the proximity between K435 and the IgA1$_{CL}$ domain. Source data are available online for this figure.

 © The Author(s)

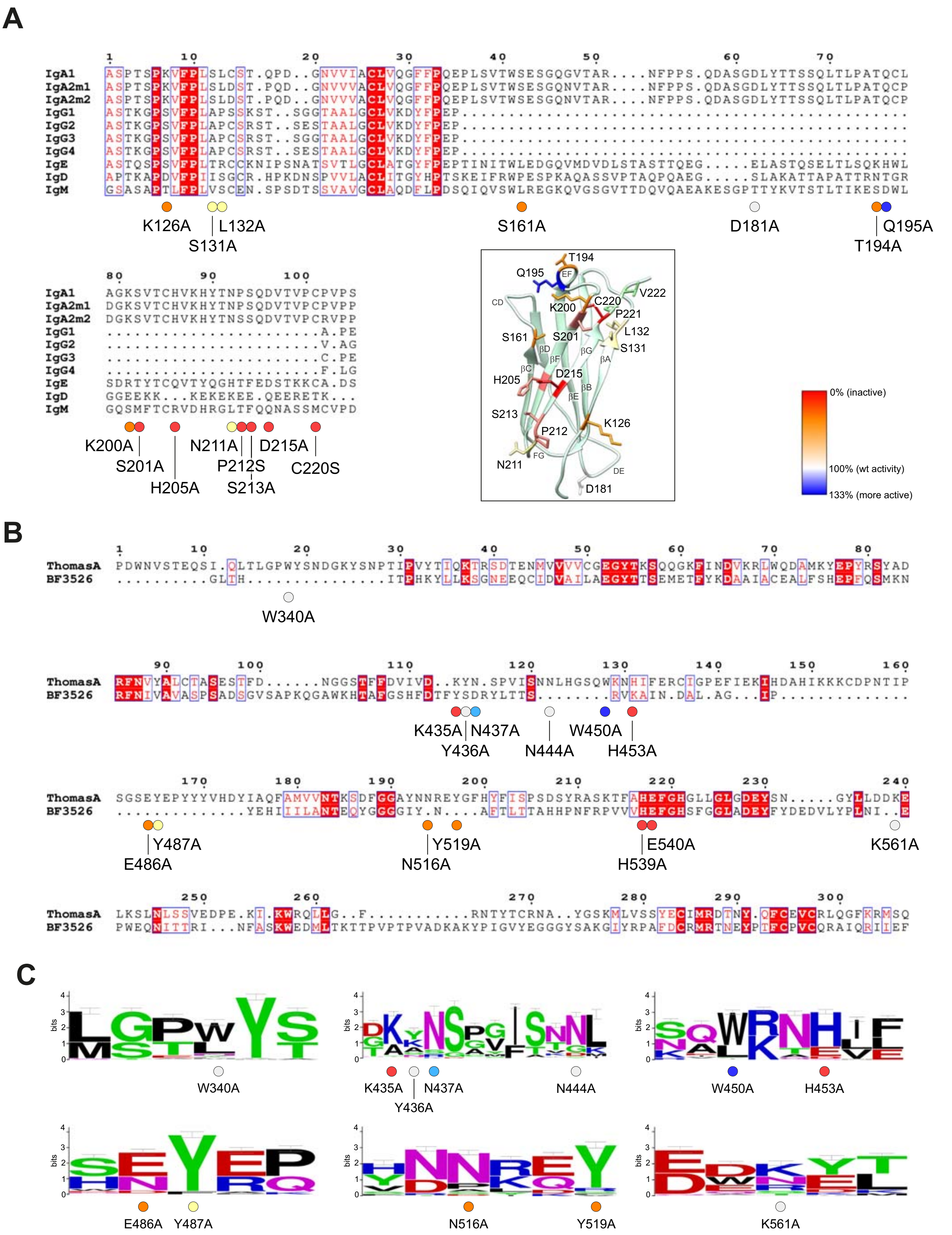
A
K126A
S131A
L132A
S161A
D181A
T194A
Q195A
K200A
S201A
H205A
N211A
P212S
S213A
D215A
C220S
0% (inactive)
100% (wt activity)
133% (more active)
B
W340A
K435A
Y436A
N437A
N444A
W450A
H453A
E486A
Y487A
N516A
Y519A
H539A
E540A
K561A
C

Figure EV5. Sequence alignment of ThomasA and BF3526 M64 peptidase domain and CH1 domain of antibody classes.

(**A**) Sequence alignment of the CH1 region of IgA1 (UniProt code P01876), IgA2m(1) (UniProt code A0A0G2JMB2), IgA2m(2) (UniProt code P01877), IgG1 (UniProt code P01857), IgG2 (UniProt code P01859), IgG3 (UniProt code P01860), IgG4 (UniProt code P01861), IgE (UniProt code P018541), IgD (UniProt code P01880) and IgM (UniProt code P01871). Inset (black square) shows a cartoon representation of the $IgA1_{CH1}$ domain within the AF3 model of $ThomasA^{323\text{-}878}$-$IgA1_{CH1}$ complex. Residues are colored according to the relative activity observed in alanine-scanning experiments. (**B**) Structural alignment of M64 peptidase domain of ThomasA and BF3526. Mutated residues from alanine-scanning experiments are highlighted with circles, with colors indicating their relative activity on a gradient scale. (**C**) Multiple sequence alignment of proteins from CL-V of our SSN generated using MUSCLE and WebLogo server. The level of conservation of selected regions that comprise the ThomasA mutants was generated with WebLogo3 (http://weblogo.threeplusone.com).
